# Scalable Hollow Fiber Adsorbents for Metal Ion Recovery via Selective In‐Pore MOF‐808 Growth under Aqueous Conditions

**DOI:** 10.1002/advs.202511437

**Published:** 2025-11-07

**Authors:** Ho Jun Lee, Cheol Lee, Ju Ho Shin, Heseong An, Go Gi Lee, Jong Suk Lee

**Affiliations:** ^1^ Department of Chemical and Biomolecular Engineering Sogang University Seoul 04107 Republic of Korea; ^2^ Department of Chemical Engineering Sunchon National University Jeollanam‐do 57922 Republic of Korea; ^3^ Industrial Materials Research Group Research Institute of Industrial Science and Technology Pohang‐si 37673 Republic of Korea; ^4^ Institute of Energy and Environmental Technology Sogang University Seoul 04107 Republic of Korea

**Keywords:** aqueous‐phase synthesis, In‐pore MOF crystallization, leaching‐resistant composites, metal ion capture, MOF‐polymer hybrid fiber sorbents

## Abstract

Selective in situ growth of metal–organic frameworks (MOFs) within polymeric supports under mild, aqueous conditions remains a synthetic challenge due to interfacial instability, uncontrolled crystallization, and MOF leaching. Here, this study reports a binding‐assisted strategy for the selective in‐pore growth of MOF‐808 within polyacrylonitrile (PAN)/polyvinyl pyrrolidone (PVP) hollow fibers at 30 °C. Alkaline hydrolysis of PAN introduces anchoring sites for zirconium clusters, while ethanol‐assisted solvation promotes MOF crystallization under ambient conditions. The spatial distribution and surface charge of hydrolyzed PVP suppress MOF nucleation on the outer surface, enabling uniform in‐pore growth with 34 wt.% loading and > 99% retention after ultrasonication. Post‐synthetic functionalization with ethylenediaminetetraacetic acid (EDTA) imparts a strong affinity for Pb^2+^, Ni^2+^, and Co^2+^ ions. The EDTA‐modified composite exhibits a 2.5‐fold increase in Pb^2+^ adsorption kinetics compared to physically blended counterparts. A modularized 105 cm fiber unit effectively treats 1 L of a mixed‐metal solution (10 ppm each), underscoring the scalability and process compatibility of this approach. This work demonstrates a mild, scalable, and leaching‐resistant route for fabricating MOF‐polymer hybrid sorbents through spatially controlled in‐pore crystallization, offering a robust platform for water treatment and metal recovery applications.

## Graphical abstract

Ethanol‐assisted solvation enables in situ crystalization of MOF‐808 within PAN/ PVP hollow fibers under mild aqueous conditions, while the spatial distribution of hydrolyzed PVP directs selective in‐pore growth. The resulting EDTA‐functionalized composite offers excellent structural stability and metal ion uptake, providing a scalable, leaching‐resistant architecture for MOF‐polymer hybrid sorbents.

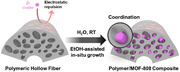

## 1 Introduction

The rapid expansion of battery manufacturing and electroplating industries has led to a significant increase in wastewater containing toxic and valuable metal ions.^[^ ^1^ ^]^ Among various heavy metal ions, Pb^2+^, Ni^2+^, and Co^2+^ are of particular concern due to their industrial relevance and toxicity.^[^ ^2^, ^3^ ^]^ Pb^2+^ is a well‐known neurotoxin commonly released from battery production and pigment manufacturing.^[^ ^3^ ^]^ Ni^2+^ and Co^2+^, which are frequently found in electroplating and alloy industries, are also classified as critical metals due to their strategic importance in energy storage and electronics.^[^ ^4^ ^]^ Consequently, the need for effective and sustainable wastewater treatment technologies has become increasingly urgent.^[^ ^5^, ^6^, ^7^ ^]^ Conventional chemical separation methods, such as precipitation and solvent extraction, often suffer from low efficiency when treating dilute metal ion solutions and produce substantial secondary waste, limiting their industrial applicability.^[^ ^8^, ^9^, ^10^ ^]^ In contrast, adsorption‐based processes have emerged as a promising alternative, offering broad concentration adaptability, low operating costs, high removal capacities, and facile regeneration.^[^ ^11^, ^12^, ^13^ ^]^

A variety of adsorbent materials, including polymers,^[^ ^14^, ^15^ ^]^ activated carbon,^[^ ^16^, ^17^ ^]^ biosorbents,^[^ ^18^, ^19^ ^]^ and metal–organic frameworks,^[^ ^20^, ^21^ ^]^ have been developed for metal ion capture. Among these, MOF‐based absorbents exhibit notable advantages due to their exceptionally high surface areas, tunable pore structures, and versatile chemical functionality.^[^ ^22^, ^23^, ^24^ ^]^ However, many MOFs display limited water stability, restricting their application in aqueous environments. Within the zirconium‐MOF family, MOF‐808 stands out as a promising candidate for wastewater treatment due to its superior chemical robustness and large surface area. MOF‐808 is a zirconium‐based MOF composed of Zr_6_O_4_(OH)_4_ secondary building units (SBUs) coordinated with benzene‐1,3,5‐tricarboxylic acid (BTC) linkers. It forms a highly porous structure featuring large mesoporous cages (18 Å), which offer high surface area and accessible coordination sites for functionalization.^[^ ^25^ ^]^ Recent studies have demonstrated that functionalization of MOF structures with chelating agents significantly enhances metal ion adsorption performance, even under dilute conditions.^[^ ^26^, ^27^, ^28^ ^]^ In particular, ethylenediaminetetraacetic acid (EDTA)‐functionalized MOF‐808 has shown outstanding affinity for a variety of metal ions at low concentration.^[^ ^29^ ^]^

While MOFs are often mobilized into pellets or packed beds to achieve high adsorbent loading per unit volume,^[^ ^30^, ^31^ ^]^ such configurations typically suffer from high pressure drops and slow adsorption kinetics, increasing operational costs and hindering large‐scale implementation.^[^ ^32^, ^33^ ^]^ Fiber‐based sorbents, incorporating MOF particles within a polymer matrix, represent an attractive alternative, offering low pressure drops, fast mass transfer, and a small footprint.^[^ ^34^, ^35^, ^36^ ^]^ For example, Ji et al.^[^ ^37^ ^]^ reported a polymer/inorganic fiber composite fabricated by dispersing zeolite particles in a spinning dope solution for heavy metal removal. Although blending MOFs into polymers prior to fiber spinning is a simple and scalable method, it often results in particle agglomeration and pore blockage, leading to reduced adsorption performance.^[^ ^38^, ^39^ ^]^ In contrast, in situ growth of MOFs within a polymer matrix enhances particle distribution uniformity and mitigates pore blockage, thereby improving adsorption efficiency.^[^ ^40^, ^41^ ^]^ However, the synthesis of water‐stable zirconium‐based MOFs, such as MOF‐808, conventionally requires high temperatures and organic solvents (e.g., DMF),^[^ ^42^, ^43^, ^44^ ^]^ making direct integration with polymer matrices challenging. Although recent advances have reported water‐based, low‐temperature synthesis routes for Zr‐based MOFs in the bulk phase,^[^ ^45^, ^46^ ^]^ the in situ growth of MOF‐808 within polymeric substrates under such mild conditions has not yet been achieved. Moreover, successful fabrication of polymer/MOF composites by in situ growth critically depends on establishing strong interactions between the polymer matrix and the growing MOF; otherwise, MOF particles may not nucleate within the polymer pores or may be easily leached during operation. Furthermore, achieving selective MOF growth within internal porous structures is inherently difficult due to diffusion limitations, often resulting in undesired MOF deposition on external surfaces.^[^ ^47^, ^48^, ^49^ ^]^

In this study, we demonstrate the efficient capture of metal ions using uniformly dispersed MOF‐808 nanofillers grown selectively within the porous structure of PAN hollow fibers. This was achieved by transforming the PAN substrate into a hybrid metal ion absorbent via in situ MOF‐808 growth under mild, water‐based conditions. Alkaline treatment of the PAN fibers introduced surface‐functional groups that served as coordination anchors for zirconium clusters, thereby preventing MOF leaching during operation. The incorporation of polyvinylpyrrolidone (PVP) into the polymer dope solution enabled selective in‐pore growth of MOF‐808 crystallization by modulating the internal pore environment. Ethanol‐induced solvation facilitates MOF crystallization within the substrate under mild conditions. Following MOF growth, the composites were functionalized with EDTA to enhance metal ion affinity. Finally, the performance and scalability of the sorbent were evaluated through modularization and dynamic breakthrough experiments to assess industrial applicability.

## 2 Results and Discussion

### 2.1 In Situ Growth Strategy for MOF‐808 Hybridization Within Polymeric Hollow Fibers

**Figure 1:**
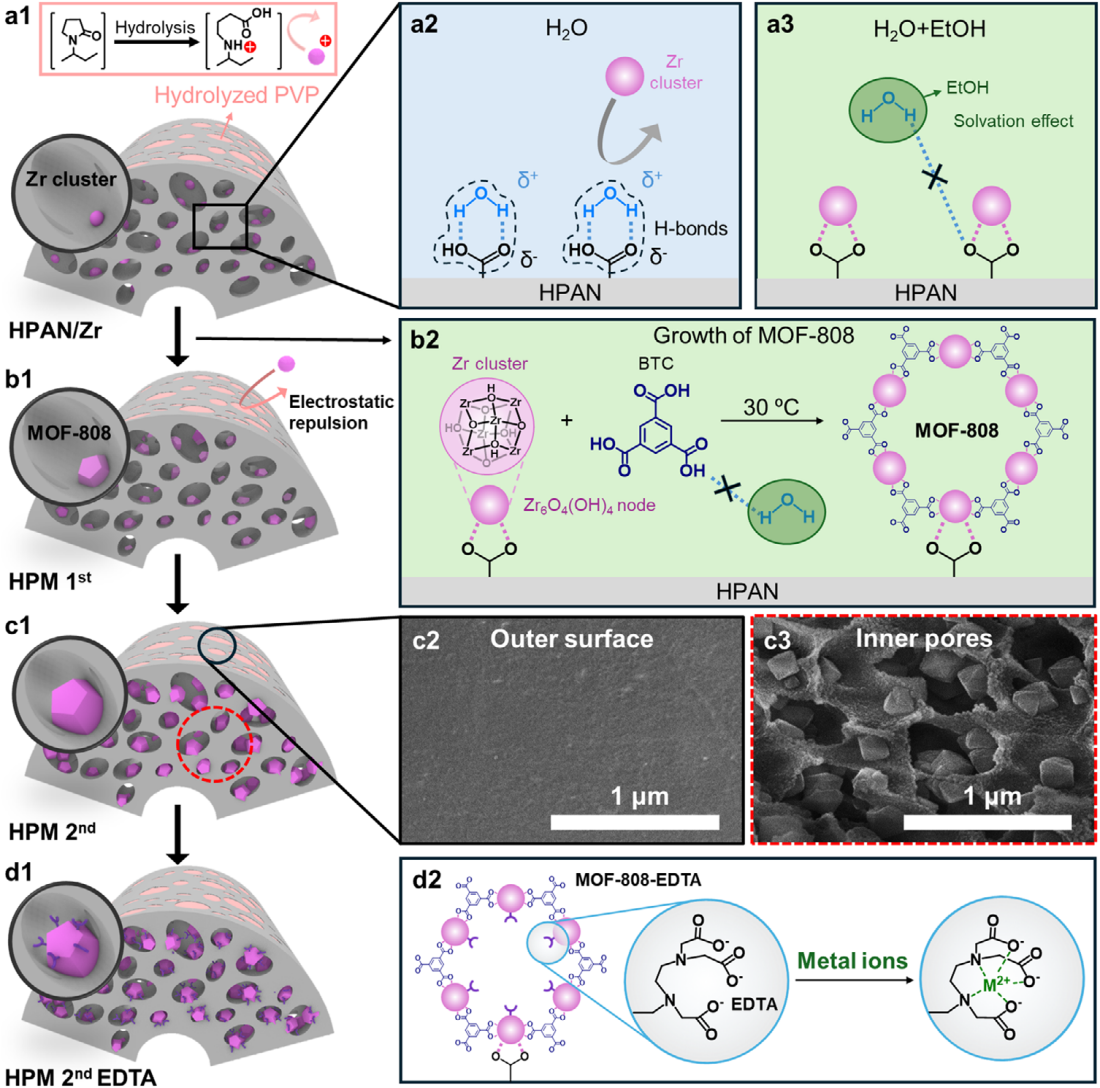
Schematic illustration of the HPAN/MOF‐808 hollow fiber composite and the fabrication strategy; ethanol‐induced solvation promoted MOF‐808 formation within HPAN under mild conditions, while spatial distribution and hydrolysis of PVP guided selective in‐pore growth via electrostatic repulsion. a1) Selective in‐pore seeding of zirconium clusters into HPAN; a2) seeding in H_2_O only and a3) seeding in a H_2_O/EtOH mixture to induce solvation‐enhanced coordination. b1, 2) Schematic of the first in situ MOF‐808 growth step within the HPAN substrate. c1) Schematic of the secondary MOF‐808 growth; c2) SEM image of the outer surface and c3) cross‐sectional SEM image of the resulting HPM 2nd composite. d1) Post‐synthetic EDTA functionalization of the HPM 2nd composite; d2) schematic of EDTA chelation with metal ions. Abbreviations: HPAN (hydrolyzed PAN), HPAN/Zr (HPAN with zirconium cluster), HPM 1st (HPAN/MOF‐808 after first growth), HPM 2nd (HPAN/MOF‐808 after secondary growth), HPM 2nd EDTA (HPM 2nd after EDTA functionalization).

Our strategy for transforming polymeric hollow fibers into hybrid metal ion absorbents via in situ MOF‐808 growth is illustrated in **Figure** **1**. A key advantage of this approach is its compatibility with water‐based, low‐temperature synthesis, which allows direct MOF growth within a polymeric matrix. Initially, carboxylic acid groups were introduced onto the PAN hollow fiber surface through alkaline hydrolysis of the nitrile (C≡N) groups using sodium hydroxide (NaOH),^[^ ^50^ ^]^ simultaneously inducing partial hydrolysis of the embedded polyvinylpyrrolidone (PVP). The resulting hydrolyzed PAN (HPAN) was then immersed in a zirconium precursor solution, enabling zirconium clusters to form and coordinate with the carboxyl groups (Figure 1a1). The addition of ethanol (EtOH) promoted a solvation effect, weakening hydrogen bonding between COOH and H_2_O and thereby facilitating the coordination of Zr clusters to HPAN (Figure 1a2,a3). In the subsequent first growth step (Figure 1b1), MOF‐808 seeds nucleated from these anchored Zr clusters. The presence of EtOH further improved loading efficiency by enhancing both the solvation environment (Figure 1b2) and trimesic acid (BTC) solubility (Figure S1, Supporting Information).

A secondary growth step (Figure 1c1) was employed to increase MOF‐808 loading and crystallinity while maintaining selective in‐pore growth (Figure 1c2,c3). This selectivity is attributed to differences in surface vs. internal composition of the substrate—primarily due to the distribution and hydrolysis of PVP, which introduces negative charges that repel Zr precursors on the outer surface but favor coordination inside the pores. Finally, the in‐pore‐grown MOF‐808 was functionalized with EDTA, imparting high affinity for metal ions and completing the formation of the hybrid fiber sorbent (Figure 1d1,d2).

### 2.2 Fabrication of Porous PAN Hollow Fiber Substrates

Porous PAN hollow fiber supports were fabricated using a dry‐jet/wet‐quench spinning process, with detailed spinning parameters and dope compositions summarized in Table S1 (Supporting Information). The design goal was to produce fibers with an interconnected pore structure while minimizing macrovoid formation. To achieve this, polyvinylpyrrolidone (PVP), a water‐soluble polymer additive commonly employed to promote pore interconnectivity during nonsolvent‐induced phase separation,^[^ ^51^, ^52^ ^]^ was incorporated at a high concentration (7 wt.%) into the dope solution. This ensured sufficient PVP presence within the PAN matrix, which later facilitated the selective in‐pore growth of MOF‐808 (further discussed in a subsequent section). The resulting hollow fibers featured a thick‐wall structure with an outer diameter (OD) of 0.97 µm and an inner diameter (ID) of 0.29 µm (Figure S2a, Supporting Information). Cross‐sectional SEM images confirmed the formation of a highly interconnected porous network (Figure S2c,d, Supporting Information), and the fiber exhibited a high porosity of 83.7% with a tensile modulus of 6.3 MPa, validating its suitability as a sorbent substrate (Figures S5 and S6, Supporting Information).^[^ ^53^ ^]^

### 2.3 Optimization of Hydrolysis and MOF‐808 Growth Conditions

**Figure 2:**
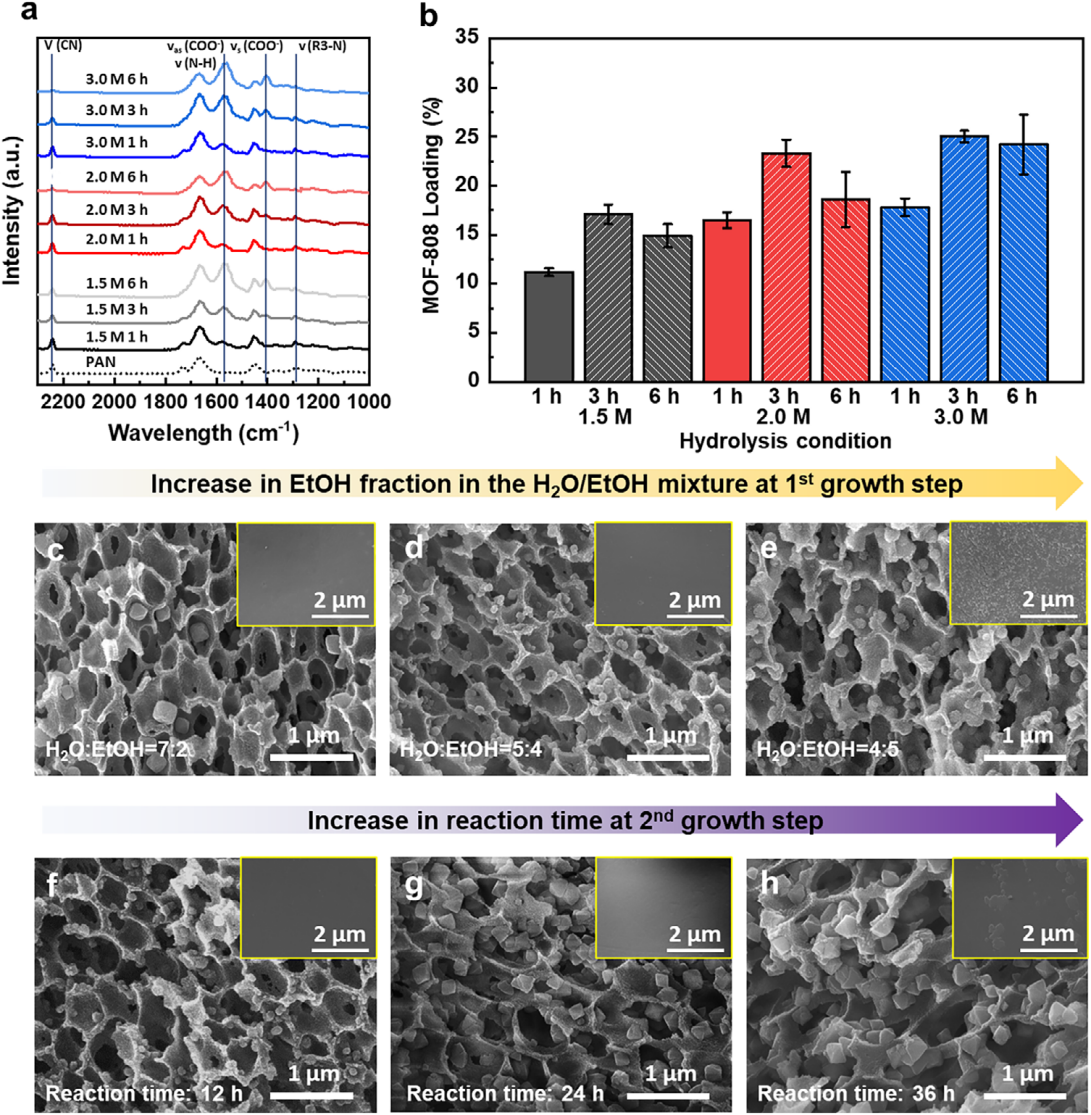
Optimization of hydrolysis and growth conditions. a) FT‐IR spectra of PAN and HPAN as a function of NaOH concentration and hydrolysis time. b) MOF‐808 loading amounts in HPAN depending on NaOH concentration and hydrolysis time. Data are shown as mean ± SD (n = 3). c–e) Cross‐sectional SEM images of composites after the first growth step with varying EtOH fractions in the H_2_O:EtOH mixture. Inset images show the outer surface SEM images: (c) HPM 1st (7:2), (d) HPM 1st (5:4), and (e) HPM 1st (4:5). f–h) Cross‐sectional SEM images of composites after the secondary growth as a function of growth time. Inset images show the outer surface SEM images: (f) HPM 2nd 12 h, (g) HPM 2nd 24 h, and (h) HPM 2nd 36 h.

The hydrolysis behavior of PAN/PVP hollow fibers was investigated to identify optimal conditions for MOF‐808 in situ growth. FT‐IR spectra of HPAN under various conditions are shown in **Figure** **2**a. The pristine PAN substrate exhibited characteristic peaks at 2242 cm^−1^ and 1453 cm^−1^, corresponding to C≡N stretching and C‐H bending vibrations, respectively.^[^ ^54^ ^]^ Peaks at 1667 cm^−1^ (C = O stretching of amide) and 1287 cm^−1^ (C‐N stretching of tertiary amine) confirmed the presence of PVP in the matrix.^[^ ^55^ ^]^ Comparative spectra of flat‐sheet membranes (FS‐PAN vs. FS‐PAN/PVP) further verified that these peaks originated from PVP, as they were absent in FS‐PAN (Figure S3, Supporting Information).

Upon hydrolysis, nitrile groups in PAN were progressively converted into amide and carboxylic groups.^[^ ^50^ ^]^ With increasing NaOH concentration and hydrolysis time, the intensity of the C≡N peak (2242 cm^−1^) decreased, while new peaks at 1571 cm^−1^ (N‐H bending of amide and asymmetric COO^−^ stretching) and 1401 cm^−1^ (symmetric COO^−^ stretching) emerged (Figure 2a).^[^ ^56^ ^]^ Simultaneously, PVP underwent base‐induced ring‐opening,^[^ ^57^, ^58^ ^]^ as evidenced by the gradual disappearance of the tertiary amine peak at 1291 cm^−1^. A similar trend was observed in FS‐PAN/PVP membranes (Figure S3, Supporting Information), supporting the reaction of PVP.

The amount of MOF‐808 incorporated after the first growth step was analyzed as a function of hydrolysis conditions (Figure 2b). MOF loading increased with hydrolysis concentration due to the greater availability of coordination sites for zirconium clusters. Regarding hydrolysis time, MOF loading increased up to 3 h but declined at 6 h. This reduction is attributed to over‐hydrolysis, which compromised the integrity of HPAN and disrupted zirconium coordination. Weight loss analysis following 48 h solvent immersion showed a 5.3% increase in degradation between 3 and 6 h hydrolysis, compared to a 1.6% increase from 1 to 3 h (Figure S4, Supporting Information), confirming the structural instability at extended hydrolysis durations.

Mechanical properties and porosity of HPAN (hydrolyzed for 3 h) were also evaluated as a function of NaOH concentration (Figures S5 and S6, Supporting Information). While porosity slightly decreased from 83.7% to 78.7% and elongation at break dropped from 9.7% to 5.6%, tensile strength increased from 6.3 to 7.0 MPa―likely due to polymer chain rigidification via hydrogen bonding.^[^ ^59^, ^60^ ^]^ These modest changes were deemed acceptable in light of the substantial improvement in MOF‐808 loading. Therefore, 3.0 M NaOH and 3 h hydrolysis were selected as optimal.

To further enhance MOF‐808 growth under mild, water‐based conditions, EtOH was introduced as a cosolvent to promote solvation effects. EtOH weakens hydrogen bonding between water and trimesic acid (BTC), thereby facilitating coordination between zirconium clusters and BTC ligands (Figure S1, Supporting Information).^[^ ^61^ ^]^ A similar mechanism is proposed wherein EtOH weakens hydrogen bonding between the COOH groups of HPAN and water molecules, thereby enhancing the coordination affinity toward Zr clusters and promoting MOF growth within the HPAN matrix (Figure S1, Supporting Information). In bulk synthesis of MOF‐808, increasing EtOH content reduced particle size, indicating accelerated nucleation (Figure S7, Supporting Information).^[^ ^62^, ^63^ ^]^ However, when the H_2_O:EtOH ratio reached 4:5, excessive nucleation led to particle agglomeration (Figure S7d, Supporting Information). SEM images of hollow fiber composites prepared with varying EtOH content (Figure 2c–e; Figure S8, Supporting Information) revealed increasing MOF formation with higher EtOH fraction. The TGA residue under oxidative conditions reflects the amount of MOF incorporated within the substrate and is commonly used for estimating MOF loading.^[^ ^64^, ^65^ ^]^ The TGA results (Figure S9, Supporting Information) align with the SEM observations. However, at an H_2_O:EtOH ratio of 4:5, MOF particles showed surface agglomeration. Thus, a 5:4 ratio was selected as optimal, balancing high loading and in‐pore selectivity.

After successful MOF‐808 incorporation during the first growth, a secondary growth step was performed to further enhance loading and crystallinity. This step decouples nucleation and crystal growth, allowing for improved crystal development while minimizing unwanted secondary nucleation.^[^ ^66^, ^67^ ^]^ Conventional seeding methods (e.g., vacuum filtration,^[^ ^68^ ^]^ spray coating,^[^ ^69^ ^]^ and surface growth^[^ ^70^ ^]^) are ineffective for internal pores, but in our system, pre‐formed MOF‐808 from the first growth served as initial seeds. To ensure gradual crystal growth and avoid new nucleation during the secondary growth, the concentrations of both the metal precursor and EtOH were reduced by half. SEM images (Figure 2f–h; Figure S10, Supporting Information) showed progressive MOF‐808 growth with increasing secondary growth time. TGA confirmed increased MOF‐808 content: 24.9%, 34.6%, 41.0%, and 45.1% at 12, 24, 36, and 48 h, respectively (Figure S11, Supporting Information). However, after 36 h, surface‐grown agglomerates were observed (Figure 2h; Figure S10, Supporting Information), indicating that 24 h is the optimal secondary growth time to achieve high loading and maintain in‐pore selectivity.

### 2.4 Structural, Chemical, and Physical Characterization at each Fabrication Step

**Figure 3:**
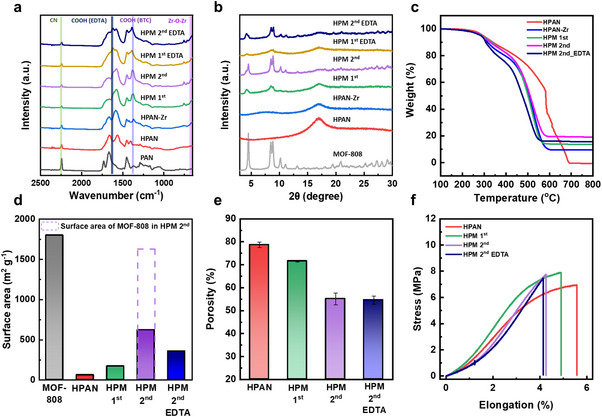
Structural and chemical characterization of HPAN and composites at each fabrication step. a) FT‐IR spectra of PAN, HPAN, and the composites. b) XRD patterns of MOF‐808, HPAN, and the composites. c) TGA curves of HPAN and the composites. d) BET surface areas of MOF‐808, HPAN, and the composites. e) Porosity changes during MOF growth and EDTA modification. Data are shown as mean ± SD (n = 3). f) Stress‐strain curves of HPAN and the composites.

The presence of functional groups and chemical interactions during each fabrication step was confirmed by FT‐IR analysis (**Figure** **3**a). The FT‐IR spectra of PAN and HPAN were discussed in the previous section. Upon zirconium coordination, HPAN‐Zr exhibited a new peak at 665 cm^−1^, corresponding to Zr‐O‐Zr stretching, confirming the formation of zirconium clusters.^[^ ^45^ ^]^ Since zirconium clusters were synthesized in the presence of formic acid, characteristic asymmetric and symmetric COO^−^ stretching vibrations of formate appeared at 1586 cm^−1^ and 1369 cm^−1^, respectively.^[^ ^45^ ^]^ Coordination between carboxylic groups and metal moieties typically induces shifts in FT‐IR peaks: a red shift in the asymmetric COO^−^ stretching and a blue shift in the symmetric COO^−^ stretching.^[^ ^71^ ^]^ The blue shift of the symmetric COO^−^ peak from 1402 cm^−1^ (HPAN) to 1423 cm^−1^ (HPAN‐Zr) indicates the formation of coordination bonds between zirconium clusters and carboxyl groups. However, the expected red shift of the asymmetric COO^−^ peak was masked by the overlapping formate signal. After MOF‐808 formation (HPM 1st and HPM 2nd), a new symmetric COO^−^ stretching peak appeared at 1384 cm^−1^, characteristic of the BTC ligand,^[^ ^72^ ^]^ confirming successful MOF‐808 growth. Upon EDTA functionalization, an additional asymmetric COO^−^ stretching peak at 1618 cm^−1^ was observed, indicating EDTA incorporation into the MOF‐808 framework.^[^ ^29^ ^]^

XRD patterns further confirmed MOF‐808 crystallinity (Figure 3b). Characteristic peaks of MOF‐808 at 4.5°, 8.5 ^°^, and 8.8° were detected in both HPM 1st and HPM 2nd composites,^[^ ^73^ ^]^ verifying the formation of the MOF‐808 framework. HPM 1st exhibited relatively weak diffraction peaks, likely due to the disordered regions and small crystallite size caused by rapid nucleation.^[^ ^74^ ^]^ After secondary growth, HPM 2nd showed markedly enhanced peak intensities, indicating improved crystallinity alongside MOF‐808 loading. EDTA functionalization slightly reduced crystallinity, likely due to partial framework distortion and zirconium‐EDTA coordination. Nevertheless, the characteristic peaks of MOF‐808 were retained, suggesting that the overall crystalline structure was preserved.

TGA analysis under an oxygen atmosphere provided further evidence of MOF‐808 incorporation (Figure 3c).^[^ ^64^ ^]^ Unlike pristine HPAN, the composites exhibited a distinct residual mass after decomposition, confirming the presence of zirconium‐based species.^[^ ^65^ ^]^ Notably, HPAN‐Zr exhibited a significantly higher residual mass (9.5%) compared to PAN‐Zr (0.0%), indicating strong interactions between Zr clusters and HPAN carboxyl groups (Figure 3c; Figure S12, Supporting Information). Since the decomposition residue of MOF‐808 is 6ZrO_2_, the MOF‐808 loading (%) was calculated using the equation: W_ZrO2_ × (M_MOF‐808_/6M_ZrO2_), where W_ZrO2_ is the residual mass (%), M_MOF‐808_ is the molecular weight of MOF‐808 (g mol^−1^), and M_ZrO2_ is the molecular weight of ZrO_2_ (g mol^−1^).^[^ ^75^ ^]^ To ensure accurate loading calculations, the MOF compositions were determined by ^1^H NMR as Zr_6_OH_4_O_4_(BTC)_2_(FA)_4.59_(OH)_0.70_(H_2_O)_0.70_ for MOF‐808 and Zr_6_OH_4_O_4_(BTC)_2_(EDTA)_2.20_(OH)_1.90_(H_2_O)_1.90_ for MOF‐808 EDTA (Figure S13, Supporting Information), indicating an EDTA loading of 33.1 wt.% in the functionalized MOF. The calculated MOF‐808 loadings were 24.5% for HPM 1st and 34.6% for HPM 2nd. After EDTA functionalization, the apparent residual mass for MOF‐808 EDTA slightly decreased due to its increased molecular weight, yielding calculated loadings of 29.0% (MOF‐808) and 37.9% (MOF‐808‐EDTA) for HPM 2nd EDTA. Importantly, the loading remained stable after 1 h of ultrasonication (Figure S14, Supporting Information), confirming that the slight decrease is attributable to binding site exchange or minor molecular weight discrepancies, not physical instability.

BET analysis revealed the evolution of the composites’ porous structure (Figure 3d; Figure S15, Supporting Information). HPAN exhibited a surface area of 60 m^2^ g^−1^, higher than the geometric surface area, indicating a porous architecture. After the first growth step, a modest increase in surface area was observed, consistent with low‐crystallinity MOF formation (Figure 3b). Secondary growth significantly increased the surface area to 619 m^2^ g^−1^, and the calculated intrinsic surface area of MOF‐808 in HPM 2nd (1621 m^2^ g^−1^) closely matched that of bulk MOF‐808 (1800 m^2^ g^−1^), suggesting successful in‐pore growth and crystallization. EDTA functionalization decreased the surface area to 355 m^2^ g^−1^, consistent with pore blocking by bulky EDTA groups.^[^ ^29^ ^]^

Porosity measurements showed a reduction from 78.7% to 71.6% after the first MOF‐808 growth, indicating partial pore filling (Figure 3e). A further significant reduction (to 55.1%) after the secondary growth confirmed increased MOF occupation within the fiber pores. The porosity values of HPM 2nd and HPM 2nd EDTA were comparable, indicating negligible loss of MOF loading during EDTA functionalization.

Mechanical properties were evaluated through tensile strength and elongation at break measurements (Figure 3f). Incorporation of MOF‐808 decreased flexibility but enhanced tensile strength, suggesting strong interactions between the substrate and the MOF.^[^ ^76^, ^77^ ^]^ Secondary growth further reduced elongation due to increased MOF loading, while EDTA functionalization had minimal additional impact. Despite reduced flexibility, the final composite (HPM 2nd EDTA) retained sufficient mechanical integrity for modular assembly (Figure S16, Supporting Information).

### 2.5 Morphologies of the Composites

**Figure 4:**
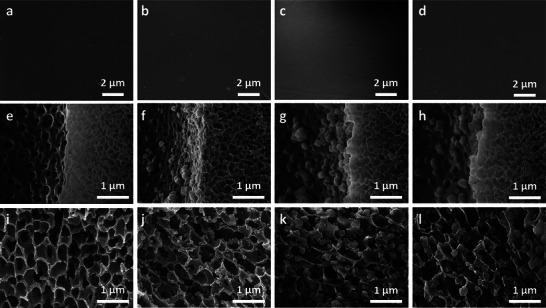
Morphological evolution of HPAN and composites at each fabrication stage a–d) Outer surface SEM images of (a) HPAN, (b) HPM 1st, (c) HPM 2nd, and (d) HPM 2nd EDTA. e–h) Cross‐sectional SEM images of the intermediate layer of (e) HPAN, (f) HPM 1st, (g) HPM 2nd, and (h) HPM 2nd EDTA. i–l) Cross‐sectional SEM images of the inner layer of (i) HPAN, (j) HPM 1st, (k) HPM 2nd, and (l) HPM 2nd EDTA.

**Figure** **4** presents SEM images of HPAN and the corresponding composites, highlighting their morphological evolution during the MOF‐808 growth and functionalization processes. On the outer surfaces of HPM 1st, HPM 2nd, and HPM 2nd EDTA, MOF‐808 particles were scarcely observed (Figure 4b–d), suggesting that MOF formation on the external surface was effectively suppressed during in situ growth. This selective in‐pore crystallization is attributed to the influence of PVP, which predominantly remains near the outer surface, as nonsolvent‐induced phase separation proceeds radially inward from the exterior, entrapping PVP molecules in the outer region of the fiber.^[^ ^78^ ^]^ Upon hydrolysis, PVP undergoes a ring‐opening reaction (Figure 1a1), generating secondary amine groups that carry positive charges.^[^ ^58^ ^]^ These positively charged groups likely repel the positively charged zirconium clusters, thereby inhibiting MOF nucleation at the fiber surface. In contrast, MOF‐808 nanoparticles with an average size of ∼150 nm were uniformly distributed within the internal pore structure after the first growth step (Figure 4j). Larger particles, however, were occasionally observed on the macrovoid surfaces (Figure 4f), which can be attributed to restricted nucleation. This is likely a result of rapid phase separation during substrate formation, leading to localized immobilization of PVP at the macrovoid interface, which further hinders MOF crystallization in those regions. It is because rapid phase separation occurring at the macrovoid surface could cause the immobilization of PVP. Following the secondary growth step, SEM images revealed notable morphological transformations. The MOF‐808 particles increased in size to ∼200 nm and adopted a well‐defined octahedral geometry (Figure 4k), indicative of the development of the intrinsic three‐dimensional crystalline structure of MOF‐808.^[^ ^72^ ^]^ These observations are in good agreement with the enhanced crystallinity observed in the XRD results (Figure 3b). Finally, SEM analysis of the EDTA‐functionalized composite (HPM 2nd EDTA) demonstrated the preservation of the MOF‐808 structure and its stable integration within the HPAN matrix (Figure 4h,l). This indicates that the EDTA modification process did not disrupt the MOF‐polymer interface, confirming the robustness of the composite under functionalization conditions.

### 2.6 Mechanistic Insights into Selective In‐Pore Growth

**Figure 5:**
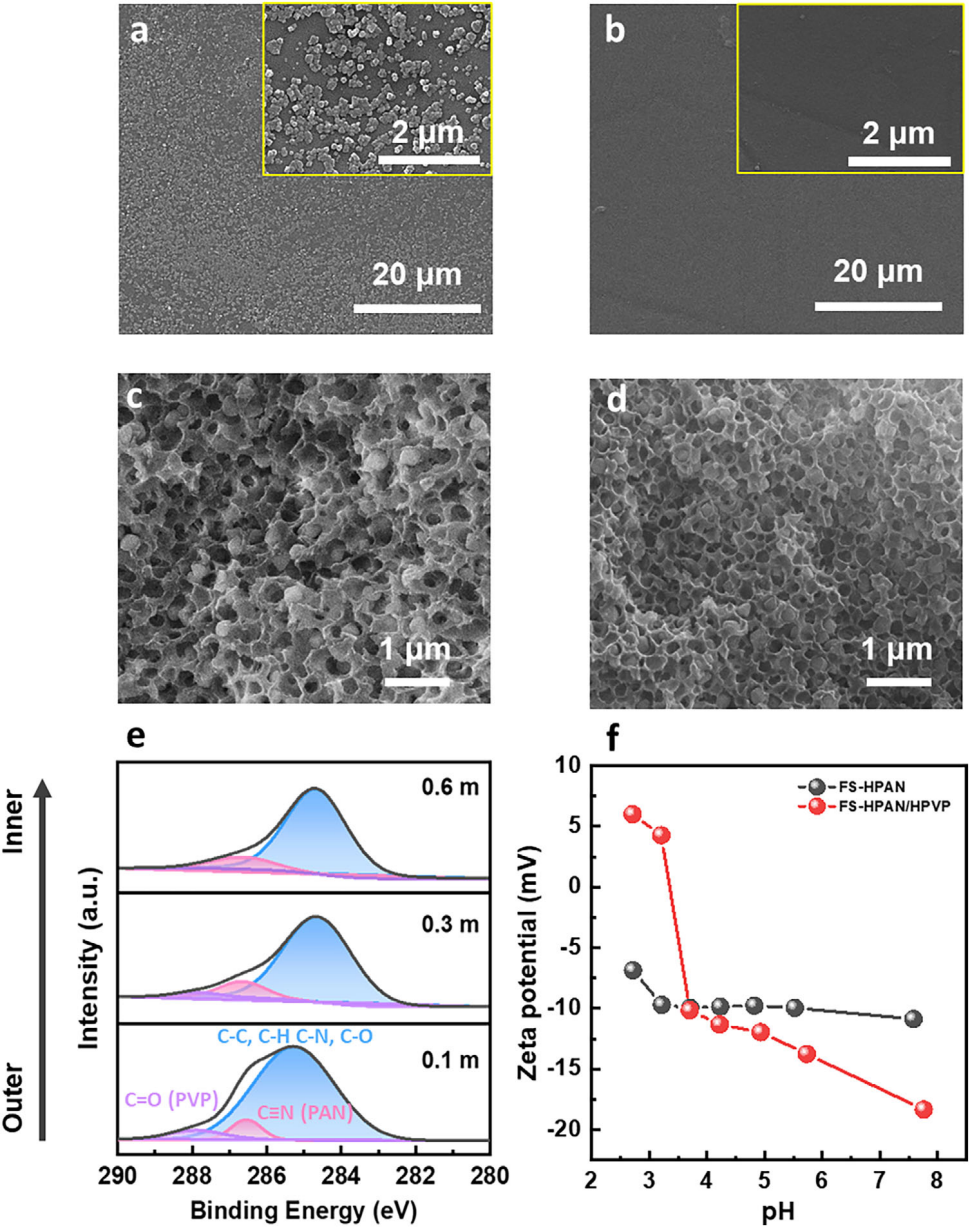
Effect of PVP on MOF‐808 growth and its role in selective in‐pore crystallization. a,b) Surface SEM images of flat‐sheets membranes after secondary MOF‐808 growth: (a) FS‐HPANM 2nd and (b) FS‐HPAN/HPVPM 2nd. c,d) Cross‐sectional SEM images of the corresponding flat sheets: (c) FS‐HPANM 2nd and (d) FS‐HPAN/HPVPM 2nd. e) XPS depth profile of the PAN hollow fiber substrate, indicating PVP distribution. f) Zeta potential of FS‐HPAN and FS‐HPAN/HPVP measured under various pH conditions.

We proposed that selective in‐pore growth of MOF‐808 is governed by two key factors: (i) electrostatic repulsion between positively charged zirconium clusters and hydrolyzed PVP, and (ii) the spatial distribution of hydrolyzed PVP within the polymer substrate. To investigate the role of PVP in directing MOF‐808 crystallization, PAN flat‐sheet membranes containing PVP (FS‐PAN/PVP) and without PVP (FS‐PAN) were fabricated and subjected to MOF‐808 growth under identical conditions. As shown in **Figure** **5**a,b, extensive MOF‐808 crystallization occurred on the surface of FS‐HPAN after secondary growth, whereas surface deposition was significantly suppressed in FS‐HPAN/HPVP, clearly demonstrating the inhibitory effect of PVP on surface MOF formation. In cross‐sectional SEM images (Figure 5c,d), both membranes exhibited in‐pore MOF‐808 growth, indicating that internal crystallization was maintained, regardless of the presence of PVP.

To further clarify the role of hydrolyzed PVP, zeta potential measurements were performed to assess the surface charge of FS‐HPAN and FS‐HPAN/HPVP membranes (Figure 5e). During alkaline hydrolysis, the lactam rings in PVP are converted to amino butyric acid moieties,^[^ ^58^ ^]^ which generally carry positive charges under acidic conditions.^[^ ^79^ ^]^ Notably, secondary amine groups, predominant in hydrolyzed PVP, are readily protonated and thus contribute to higher positive surface charges compared to primary or tertiary amines.^[^ ^80^ ^]^ Indeed, FS‐HPAN/HPVP exhibited a significantly higher positive charge than FS‐HPAN at pH values below 3.5. Given that the MOF‐808 growth solution had a pH of 1.2, strong electrostatic repulsion between the positively charged hydrolyzed PVP and zirconium clusters likely suppressed MOF nucleation and growth on the outer surface.

XPS depth profiling further elucidated the distribution of PVP within the PAN hollow fiber substrate (Figure 5f). The relative abundance of PVP, estimated from the C = O/C≡N ratio,^[^ ^81^, ^82^ ^]^ decreased markedly with etching depth (Figure 5d; Table S2, Supporting Information), confirming that PVP was concentrated near the outer surface. This distribution pattern is attributed to the dynamics of nonsolvent‐induced phase separation (NIPS) during fiber fabrication.^[^ ^78^ ^]^ Rapid phase separation at the fiber surface limited PVP mobility, leading to its immobilization in this region. In contrast, delayed phase separation in the inner pore structure allowed PVP to diffuse out more readily, resulting in a lower concentration in the internal regions. Collectively, these findings confirm that the selective in‐pore growth of MOF‐808 is enabled by the strategic localization of hydrolyzed PVP near the outer surface, where it inhibits MOF crystallization through electrostatic repulsion. Simultaneously, the lower PVP concentration in the internal pores creates a favorable environment for zirconium cluster coordination and MOF‐808 formation, thus achieving targeted in‐pore growth.

### 2.7 Metal Ion Adsorption Performance

**Figure 6:**
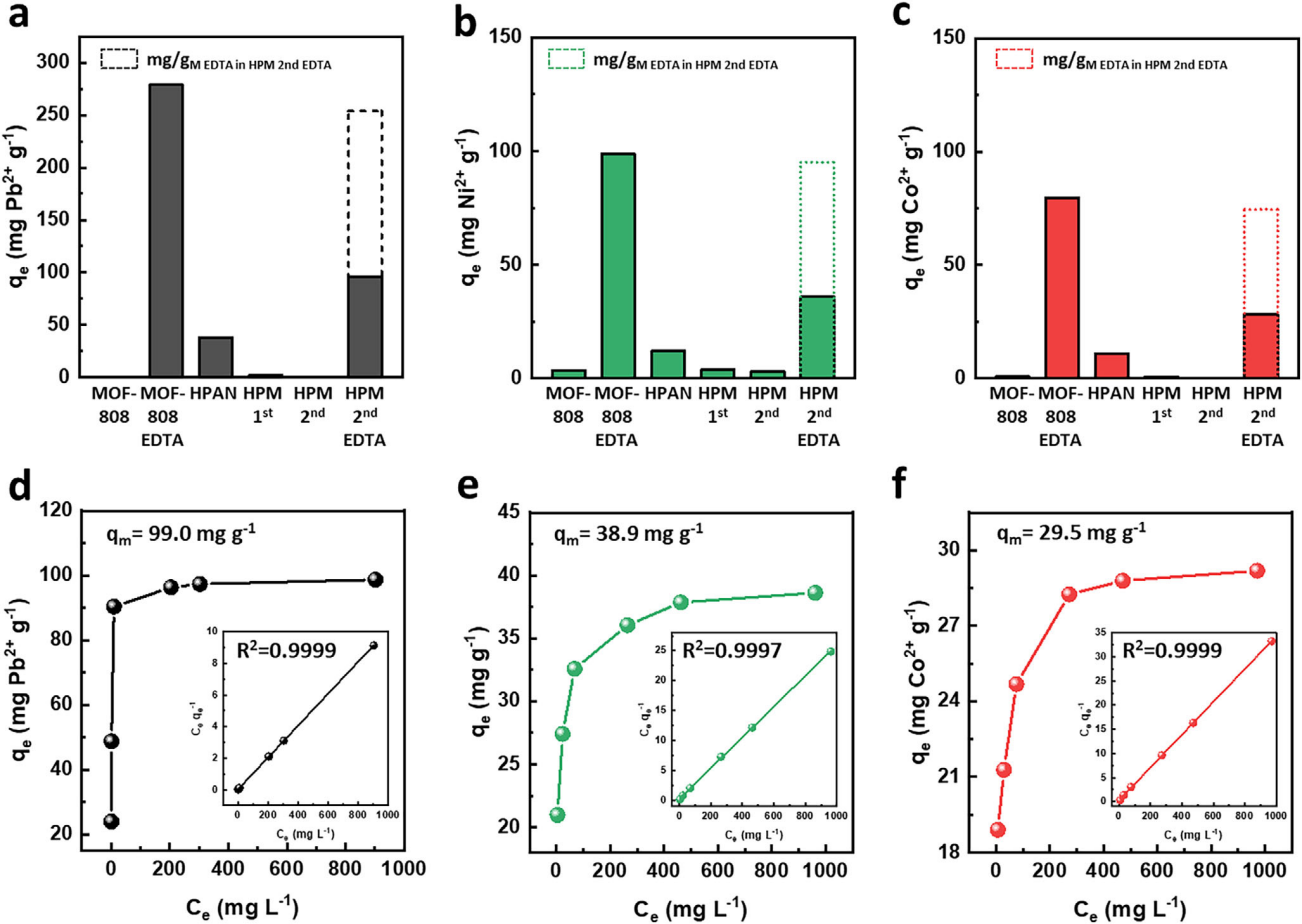
Metal ion adsorption performance at each fabrication stage and evaluation of the adsorption capacity of HPM 2nd EDTA. a–c) Equilibrium adsorption capacities of MOF‐808, MOF‐808 EDTA, HPAN, HPM 2nd, and HPM 2nd EDTA in 300 ppm metal solutions for (a) Pb^2+^, (b) Ni^2+^, and (c) Co^2+^. d–f) Adsorption isotherms of HPM 2nd EDTA at 25 °C for (d) Pb^2+^, (e) Ni^2+^, and (f) Co^2+^. Inset images show corresponding Langmuir linear fittings.

A comparative analysis of heavy metal ion adsorption capacities at each fabrication step provides valuable insights into how the structural and chemical properties of the composites influence metal capture (**Figure** **6**a–c). As expected, bulk‐synthesized MOF‐808 exhibited negligible adsorption toward all tested metal ions, reflecting its lack of chelating functionality. In contrast, EDTA‐functionalized MOF‐808 displayed markedly enhanced adsorption capacities, achieving 253.9, 95.0, and 74.5 mg g^−1^ for Pb^2+^, Ni^2+^, and Co^2+^, respectively, comparable with previously reported values.^[^ ^29^, ^44^ ^]^ These results underscore the critical role of EDTA functionalization in imparting selective metal ion affinity.

For comparison, hydrolyzed PAN (HPAN), containing carboxylic acid groups, exhibited moderate adsorption capacities of 37.9, 12.0, and 11.0 mg g^−1^ for Pb^2+^, Ni^2+^, and Co^2+^, respectively. However, the incorporation of MOF‐808 into HPAN reduced the available adsorption sites by consuming carboxylic groups through coordination with zirconium clusters, thus lowering metal ion uptake. Following EDTA modification, the resulting HPM 2nd EDTA composite exhibited significantly improved adsorption capacities of 96.3, 36.1, and 28.3 mg g^−1^ for Pb^2+^, Ni^2+^, and Co^2+^, respectively. Notably, the reduction in adsorption capacity of MOF‐808 EDTA in HPM 2nd EDTA was less than 10% compared to that of bulk‐synthesized MOF‐808 EDTA, indicating that the in situ growth strategy effectively minimizes site loss typically associated with particle agglomeration and pore blockage during polymer integration.

The adsorption isotherms of HPM 2nd EDTA for Pb^2+^, Ni^2+,^ and Co^2+^ at 25 °C are presented in Figure 6d–f. Fitting of the data using the Langmuir isotherm model yielded high correlation coefficients (R^2^ > 0.99), suggesting monolayer adsorption onto homogeneous binding sites (Table S3, Supporting Information).^[^ ^83^ ^]^ The calculated maximum adsorption capacities (q_m_) were 99.0 mg g^−1^ for Pb^2+^, 38.9 mg g^−1^ for Ni^2+,^ and 29.5 mg g^−1^ for Co^2+^. Additionally, reusability tests over four consecutive adsorption‐desorption cycles demonstrated that HPM 2nd EDTA retained ≈95% of its initial capacity (Figure S17, Supporting Information), confirming excellent stability and regeneration capability.

The effect of pH on the adsorption capacity of HPM 2nd EDTA toward Pb^2+^, Ni^2+^, and Co^2+^ was studied (Figure S18, Supporting Information). As anticipated, the adsorption capacity decreased under acidic conditions due to the protonation of EDTA functional groups—such as carboxylic acids and tertiary amines—as well as increased competition between H^+^ ions and metal cations for binding sites.^[^ ^84^ ^]^

Adsorption kinetics were further investigated to elucidate the capture mechanism. The uptake profiles of HPM 2nd EDTA over time (Figure S19, Supporting Information) were analyzed using the pseudo‐second‐order kinetic model, with fitting results summarized in Table S4 (Supporting Information). The high correlation coefficients indicate that chemisorption is the dominant adsorption mechanism (Figure S19, Supporting Information). Metal ion uptake increased rapidly during the first 3 h and gradually approached equilibrium. Pb^2+^ and Co^2+^ reached equilibrium within 6 h, whereas Ni^2+^ required ≈12 h. This delay is attributed to the higher dehydration energy of Ni^2+^, which hampers its complexation with EDTA relative to Pb^2+^ and Co^2+^.^[^ ^85^ ^]^

### 2.8 Modularization, Dynamic Adsorption Performance, and Kinetic Comparison Between In Situ Growth and Physical Blending

**Figure 7:**
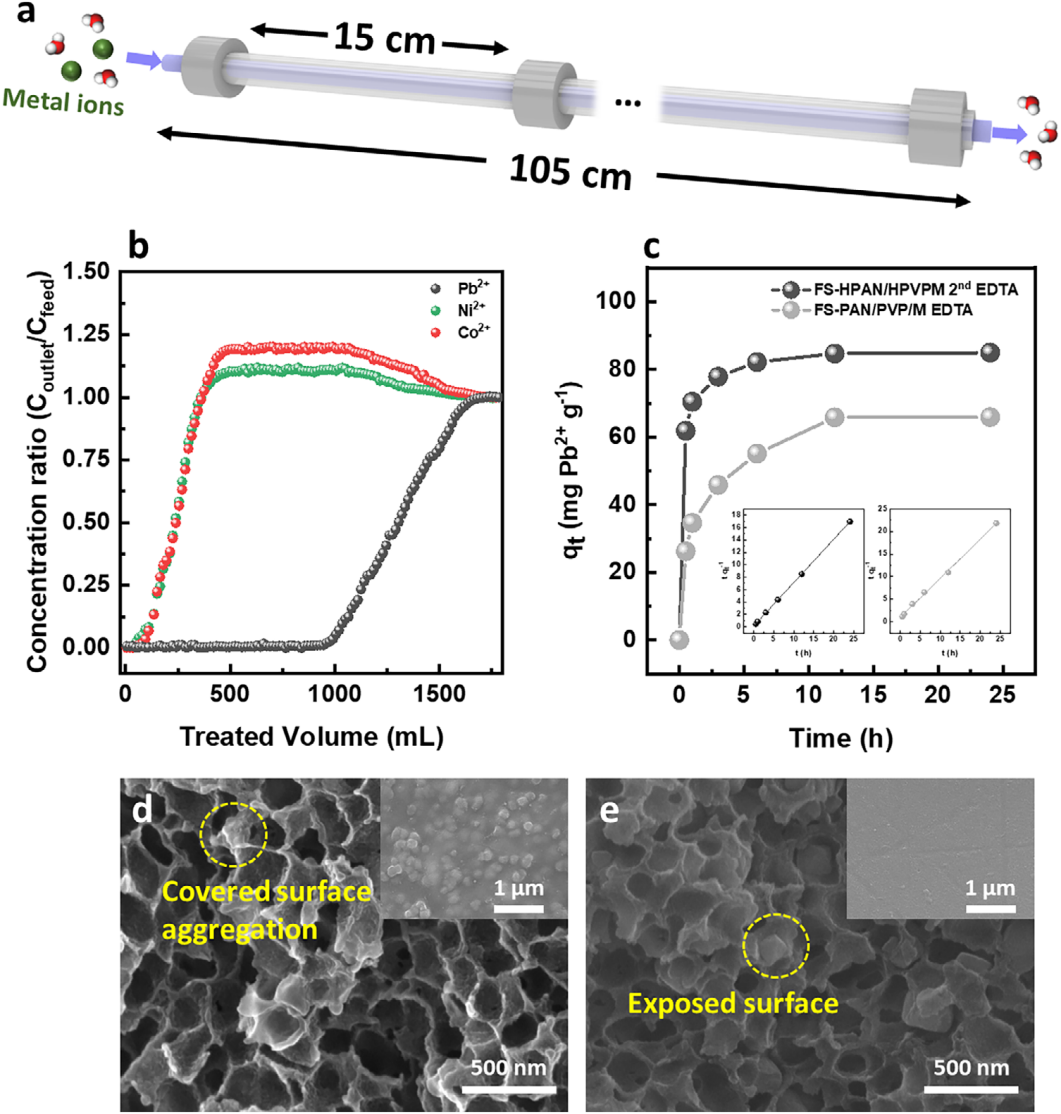
Module design and evaluation of dynamic metal ion capture performance, along with kinetic comparison between in situ growth and physical blending. a) Schematic of the hollow fiber module used for dynamic adsorption experiments. b) Breakthrough curves for Pb^2+^, Ni^2+^, and Co^2+^ under continuous flow conditions. c) Comparison of adsorption kinetics between FS‐HPAN/HPVPM 2nd EDTA (in situ growth) and FS‐PAN/PVP/M EDTA (physical blending). d,e) Cross‐sectional SEM images of the flat sheets prepared by in situ growth and physical blending; inset images show the outer surface SEM images: (d) FS‐PAN/PVP/M EDTA (e) FS‐HPAN/HPVPM 2nd EDTA.

**Figure** **7**a illustrates the design of the hollow fiber module used for dynamic metal ion capture, with additional setup details provided in Figure S20 (Supporting Information). Seven HPM 2nd EDTA hollow fibers, each 15 cm long, were assembled to create a total fiber length of 105 cm. This module was tested using a breakthrough experiment with a mixed metal ion solution (10 ppm each of Pb^2+^, Ni^2+^, and Co^2+^) to evaluate performance under continuous flow conditions (Figure 7b). During the test, the feed solution was introduced at 0.5 bar with a flow rate of 0.05 ml min^−1^, regulated by a restrictor at the outlet. The effluent was collected over time and analyzed by ICP‐OES to determine metal ion concentrations. At the early stages of operation, the outlet concentrations for all metal ions remained near zero, confirming efficient capture by the fiber module (Figure 7b). Among the three ions, Pb^2+^ showed the most delayed breakthrough, reflecting its stronger affinity toward the HPM 2nd EDTA. This result is consistent with the Langmuir isotherm analysis (Table S3, Supporting Information), which revealed higher Langmuir constants and maximum adsorption capacities for Pb^2+^ compared to Ni^2+^ and Co^2+^. Furthermore, the observed increase in Ni^2+^ and Co^2+^ outlet concentrations (exceeding unity in the normalized breakthrough curves) suggests displacement by Pb^2+^, which preferentially binds to EDTA due to its higher affinity.

To further evaluate the benefits of the in situ growth strategy, adsorption kinetics were compared using two flat‐sheet samples: FS‐HPAN/HPVPM 2nd (prepared via in situ growth, mirroring HPM 2nd) and FS‐PAN/PVP/M (prepared by physically blending MOF‐808 with PAN/PVP). After EDTA functionalization, both samples were tested for Pb^2+^ adsorption (300 ppm) using the pseudo‐second‐order kinetic model (Figure 7c; Table S5, Supporting Information). FS‐HPAN/HPVPM 2nd EDTA demonstrated superior performance, with a higher kinetic rate constant (0.8175 mg mg^−1^ min^−1^) and equilibrium adsorption capacity (86.2 mg g^−1^) compared to FS‐PAN/PVP/M EDTA, which exhibited lower values (0.3037 mg mg^−1^ min^−1^ and 69.4 mg g^−1^, respectively). These differences are directly related to the accessibility of MOF‐808 particles within the composites. SEM images of FS‐HPAN/HPVPM 2nd and FS‐PAN/PVP/M (Figure 7d,e) revealed that, in the physically blended sample, MOF‐808 particles were embedded within the polymer matrix and aggregated, reducing their exposure to the solution. This encapsulation likely impeded EDTA functionalization and limited metal ion access to active sites, resulting in diminished adsorption capacity and slower kinetics. In contrast, MOF‐808 particles in FS‐HPAN/HPVPM 2nd were more accessible due to selective in‐pore growth, enhancing both functionalization efficiency and adsorption performance. Additionally, mechanical integrity varied between the two samples. FS‐HPAN/HPVPM 2nd EDTA maintained flexibility, whereas FS‐PAN/PVP/M EDTA exhibited brittleness and reduced mechanical durability (Figure S21, Supporting Information), likely caused by particle aggregation and poor dispersion within the polymer matrix. Overall, the proposed in situ, binding‐assisted selective in‐pore growth strategy effectively prevents particle aggregation and leaching while maintaining high particle accessibility. This approach enables efficient and robust metal ion capture, underscoring its potential for practical application in modular, scalable adsorption systems.

## 3 Conclusion

In summary, we developed a new water‐based strategy for the selective in‐pore growth of MOF‐808 within polymeric hollow fibers under mild conditions. This approach enabled the fabrication of hybrid fiber sorbents with high MOF loading (34%) while effectively suppressing undesired outer surface crystallization. The resulting composites retained surface areas and metal ion adsorption capacities comparable to pristine MOF‐808, despite their integration into a polymer matrix. Importantly, the in situ grown composites demonstrated significantly enhanced adsorption kinetics. For instance, the Pb^2+^ adsorption rate constant was ≈2.5 times higher than that of composites prepared by physical blending, highlighting the superior accessibility and dispersion of MOF‐808 achieved through the in situ growth method. Mechanistic investigations confirmed that selective in‐pore crystallization is driven by the distribution and positive charge characteristics of hydrolyzed PVP, which govern MOF nucleation and growth sites. Finally, the practical potential of this approach was validated through modularization and dynamic adsorption tests. The fiber module exhibited excellent breakthrough performance under continuous mixed metal ion flow, demonstrating the scalability and applicability of this hybrid sorbent design for advanced, fiber‐based metal ion capture technologies. While MOF‐808 and PAN/PVP were selected as model systems in this study, the design principles presented here—namely, asymmetric surface charge engineering, aqueous in situ growth, and binding‐assisted confinement—may be applicable to a broader range of MOFs and polymer matrices, offering a versatile platform for future development of scalable fiber‐based adsorbents.

## Conflict of Interest

The authors declare no conflict of interest.

## Author Contributions

H.J.L. and C.L. contributed equally to this work. H.J.L. and C.L. performed conceptualization, investigation, data curation, visualization, and wrote the original draft. J.H.S. and H.A. performed data curation and visualization. G.G.L. performed validation and resources. J.S.L. performed conceptualization, validation, supervision, project administration, wrote, review and edited the final manuscript.

## Supporting information



Supporting Information

## Data Availability

The data that support the findings of this study are available from the corresponding author upon reasonable request.
