# Supplementary material for: Scalable Hollow Fiber Adsorbents for Metal Ion Recovery via Selective In‐Pore MOF‐808 Growth under Aqueous Conditions
Source: Adv Sci (Weinh). 2025 Nov 7;13(5):e11437. doi: 10.1002/advs.202511437 (PMC12850194; doi:10.1002/advs.202511437)
Supplement: Supplementary file 1 — Supporting Information [file ADVS-13-e11437-s001.docx]

**Supplementary Information**

Scalable Hollow Fiber Adsorbents For Metal Ion Recovery via Selective In-Pore MOF-808 Growth under Aqueous Conditions

*Ho Jun Lee, Cheol Lee,* *Ju Ho Shin, Heseong An, Go Gi Lee, Jong Suk Lee**

H. J. Lee, C. Lee, J. H. Shin, J. S. Lee

Department of Chemical and Biomolecular Engineering, Sogang University, Seoul 04107, Republic of Korea

E-mail: jongslee@sogang.ac.kr

J. S. Lee

Institute of Energy and Environmental Technology, Sogang University, Seoul 04107, Republic of Korea

H. An

Department of Chemical Engineering, Sunchon National University, Jeollanam-do 57922, Republic of Korea

G. G. Lee

Industrial Materials Research Group, Research Institute of Industrial Science and Technology, Pohang-si, 37673, Republic of Korea

Keywords: In-pore MOF crystallization, aqueous-phase synthesis, MOF-polymer hybrid fiber sorbents, leaching-resistant composites, metal ion capture

**1. Materials and Methods**

**Materials**

Polyacrylonitrile (PAN, M_n_ $\approx$ 150 kDa) was purchased from SY Innovation (South Korea). Polyvinylpyrrolidone (PVP, M_n_ $\approx$ 40 kDa), dimethyl sulfoxide (DMSO), ethanol (EtOH), sodium hydroxide (NaOH), lead nitrate (Pb(NO_3_)_2_), nickel nitrate hexahydrate (Ni(NO_3_)_2_·6H_2_O), and cobalt nitrate hexahydrate (Co(NO_3_)_2_·6H_2_O) were obtained from Daejung (South Korea). Zirconium(IV) chloride (ZrCl_4_) and benzene-1,3,5-tricarboxylic acid (BTC) were purchased from Alfa Aesar (USA). Formic acid was obtained from Tokyo Chemical Industry (Japan), and ethylenediaminetetraacetic acid disodium salt (EDTA-2Na) was purchased from Sigma Aldrich (USA). All chemicals were used as received without further purification.

**PAN Hollow fiber fabrication**

PAN hollow fiber supports were fabricated using a dry-jet/wet-quench spinning process. The spinning parameters and dope composition are summarized in **Table S1**. The spinning dope, composed of PAN (18 wt%), PVP (7 wt%), and DMSO (75 wt%), was mixed on a roller until a homogenous solution was obtained. Then, the dope solution and the bore fluid (DMSO:H_2_O=9:1) were loaded into a syringe pump, co-extruded through a spinneret, and quenched in a water bath to induce phase separation. The resulting PAN/PVP hollow fibers were collected on a take-up drum, cut into 30 cm lengths, and soaked in deionized water for 3 days with daily water replacement. Fibers were stored in fresh deionized water until further use.

**Flat sheet fabrication (FS-PAN, FS-PAN/PVP)**

PAN flat sheets were prepared via a non-solvent induced phase separation (NIPs) method. For the PAN-only flat sheet (FS-PAN), PAN powder (18 wt%) was dissolved in DMSO (82 wt%) at 70 ^o^C and stirred until a homogeneous solution formed. The solution was sonicated in a water bath for 3 h to remove air bubbles, then heated to 50 ^o^C and cast onto a preheated glass plate (50 ^o^C) using a casting knife. The cast film was immediately immersed in a water quench bath (25 ^o^C) to induce phase separation. The resulting membrane was rinsed with deionized water and stored in deionized water until use. For the PAN/PVP flat sheet (FS-PAN/PVP), PAN (18 wt%) and PVP (7 wt%) were dissolved in DMSO (75 wt%), and processed using the same procedure.

**Hydrolysis of substrates**

Each PAN substrate was treated with NaOH solutions of varying concentrations (1.5 M, 2.0 M, and 3.0M) for different durations (1 h, 3 h, and 6 h). After hydrolysis, the samples were washed several times with deionized water, soaked in a water bath for 24 h, and then freeze-dried. The resulting hollow fiber samples were denoted as x M y h HPAN, where x and y represent the NaOH concentration and hydrolysis time, respectively. The optimized condition (3.0 M NaOH for 3 h) was applied for the hydrolysis of FS-PAN and FS-PAN/PVP, and the resulting flat sheets were denoted as FS-HPAN and FS-HPAN/HPVP, respectively.

**Synthesis of HPAN-Zr and PAN-Zr**

ZrCl_4_ (0.96 g) and formic acid (FA, 13.2 g) were added to a 36 mL solution of a water/ethanol mixture (v/v = 5:4). Prepared PAN and HPAN hollow fiber substrates were immersed in this solution at 30 ^o^C for 24 h to allow zirconium cluster formation within the HPAN matrix. The samples were then washed several times with deionized water and soaked overnight in water to remove unreacted zirconium precursors. Finally, the composites were freeze-dried. To investigate the role of carboxylic acid (-COOH) groups generated through hydrolysis, both PAN-Zr and HPAN-Zr were prepared and analyzed by TGA to determine zirconium cluster loading.

**Synthesis of HPM 1st**

ZrCl_4_ (0.96 g) and FA (13.2 g) were dissolved in 36 mL of water/ethanol mixture (v/v = 5:4). HPAN hollow fibers (3.0 M, 3 h) were immersed in this solution at 30 ^o^C for 24 h. Subsequently, BTC ligands were added, and the fibers were incubated for an additional 48 h to facilitate the in-situ growth of MOF-808. The resulting composites were washed several times with deionized water and soaked overnight in an H_2_O/EtOH mixture (1:2) to remove residual precursors. The final products were freeze-dried and denoted as HPM 1st.

**Synthesis of HPM 2nd**

ZrCl_4_ (0.48 g) and FA (13.2 g) were added to 36 mL of an H_2_O/EtOH mixture (v/v = 7:2) and incubated at 30 ^o^C for 24 h to pre-form zirconium clusters. HPM 1st was then immersed in the solution, BTC ligands were introduced, and the mixture was further incubated at 30 ^o^C for 24 h to induce secondary MOF-808 growth. The composites were washed with deionized water and soaked overnight in an H_2_O/EtOH mixture (1:2) to remove unreacted species. Finally, the samples were freeze-dried and labeled HPM 2nd.

**Synthesis of HPM 2nd EDTA**

EDTA-2Na (1.86 g) was dissolved in 50 mL of water, and HPM 2nd was immersed in the solution at 60 ^o^C for 24 h to introduce EDTA into the MOF-808 structure. The resulting EDTA-functionalized composite was washed thoroughly with deionized water and soaked overnight to remove excess EDTA. The final product, denoted as HPM 2nd EDTA, was dried by freeze-drying.

**Optimization of hydrolysis conditions**

Hydrolysis conditions were optimized to create a favorable environment for MOF-808 growth while maintaining the pore structure and mechanical integrity of the PAN-based substrate. To compare MOF-808 loading under different conditions, each hydrolyzed hollow fiber sample was used as a substrate for in-situ MOF-808 growth. A precursor solution was prepared by adding 0.96 g of ZrCl_4_ and 13.2 g of FA to a 36 mL solution of an H_2_O/EtOH (v/v = 5:4). HPAN substrates were immersed in this solution at 30 ^o^C for 24 h to form zirconium clusters. Subsequently, BTC ligands were added, and the substrates were incubated for an additional 48 h to allow in-situ MOF-808 crystallization. The resulting composites were washed several times with deionized water and soaked overnight in an H_2_O/EtOH mixture (1:2) to remove unreacted precursors. Final samples were freeze-dried. MOF-808 loading was quantified via TGA. For samples hydrolyzed for 3 h, porosity and mechanical strength were also evaluated as a function of NaOH concentration to assess structural changes induced by hydrolysis.

**Optimization of first growth**

EtOH was used as an additive to regulate the nucleation rate and MOF-808 loading, while ensuring selective in-pore growth. To optimize the first growth conditions, MOF-808 loading and composite morphology were investigated as a function of EtOH content in the synthesis solution. ZrCl_4_ (0.96 g) and FA (13.2 g) were added to 36 mL of H_2_O/EtOH mixture with volume ratios of 9:0, 7:2, 5:4, and 4:5. HPAN hollow fibers (3.0 M, 3 h) were immersed in the solution at 30 ^o^C for 24 h. BTC ligands were then introduced, and the system was incubated for another 48 h. The composites were washed thoroughly with deionized water and soaked overnight in an H_2_O/EtOH mixture (1:2) to remove residual precursors. Final products were freeze-dried and denoted as HPM 1st (x:y), where x and y represent the H_2_O/EtOH volume ratio. MOF-808 loading was quantified via TGA, and morphologies were characterized by FE-SEM.

**Optimization of secondary growth**

Secondary growth was optimized to enhance crystallinity and further increase MOF-808 loading while maintaining selective in-pore formation. To improve crystal quality and prevent secondary nucleation outside the pores, the concentrations of both the metal precursor and EtOH were halved. ZrCl_4_ (0.48 g) and FA (13.2 g) were added to 36 mL of an H_2_O/EtOH solution (v/v = 7:2), which was incubated at 30 ^o^C for 24 h. The HPM 1^st^ (5:4) samples were then immersed, BTC ligands were introduced, and the solution was incubated at 30 ^o^C for 12 h, 24 h, 36 h, and 48 h. The composites were designated as HPM 2nd 12 h, HPM 2nd 24 h, HPM 2nd 36 h, and HPM 2nd 48 h, respectively. After growth, the samples were washed with deionized water, soaked overnight in an H_2_O/EtOH mixture (1:2), and freeze-dried. MOF-808 loading was evaluated via TGA, and morphology was examined by FE-SEM.

**Bulk synthesis of MOF-808 and MOF-808 EDTA**

ZrCl_4_ (0.96 g) and FA (13.2 g) were added to 36 mL of an H_2_O/EtOH mixture (v/v = 5:4) and incubated at 30 ^o^C for 24 h to form zirconium clusters. BTC ligands were then introduced, and the solution was incubated for an additional 48 h to form bulk MOF-808. The product was washed three times with an H_2_O/EtOH mixture (1:2) by centrifugation, soaked overnight in EtOH, and dried at 60 ^o^C under vacuum. For EDTA functionalization, EDTA-2Na (1.86 g) was dissolved in water (50 mL), and MOF-808 (0.10 g) was then dispersed in the solution and reacted at 60 ^o^C for 24 h. The resulting MOF-808 EDTA was washed with H_2_O by centrifugation, quenched in EtOH overnight, and dried under vacuum at 60 ^o^C.

**Synthesis of FS-HPANM 2nd and FS-HPAN/PVPM 2nd**

The same optimized conditions—hydrolysis (3 M, 3 h), first growth (H_2_O:EtOH = 5:4), and secondary growth (24 h)—were applied to the FS-PAN and FS-PVP/PVP flat sheet substrates.

**Simple blending of PAN/PVP with MOF-808 (FS-PAN/PVP/M)**

To compare the adsorption behavior of a physically blended polymer-MOF-808 composite with that of the in-situ grown system, a flat-sheet composite (FS-PAN/PVP/M) was fabricated by blending MOF-808 with a PAN/PVP matrix. PAN powder (18 wt%) and PVP (7 wt%) were dissolved in DMSO (68.7 wt%) containing dispersed MOF-808 (6.3 wt%) at 70 ^o^C to form a homogeneous solution. The solution was sonicated in a water bath for 3 h to remove air bubbles, then heated to 50 ^o^C and cast onto a preheated glass plate using a casting knife (both at 50 ^o^C). The cast film was immediately immersed in a water quench bath (25 ^o^C) to induce phase separation. The resulting substrate was rinsed with deionized water and freeze-dried.

**Synthesis of FS-PAN/PVP/M EDTA and FS-HPAN/HPVPM 2nd EDTA**

EDTA-2Na (1.86 g) was dissolved in 50 mL of water. FS-PAN/PVP/M (0.3 g) or FS-HPAN/HPVPM 2nd was immersed in the solution at 60 ^o^C for 24 h to introduce EDTA functionality. The resulting composites were washed thoroughly with EtOH and H_2_O and then freeze-dried. The products were denoted as FS-PAN/PVP/M EDTA and FS-HPAN/HPVPM 2nd EDTA, respectively.

**Supplementary characterizations**

Metal ion concentrations after adsorption experiments were analyzed using Inductively Coupled Plasma Optical Emission Spectroscopy (ICP-OES, ICP-5800, Agilent). Morphologies of the fiber sorbents and MOF-808 particles were observed using field-emission scanning electron microscopy (FE-SEM, JSM-7100F, JEOL). Surface charges of hollow fibers were analyzed using streaming zeta potential measurements (Surpass 3, Anton Paar). X-ray diffraction (XRD) patterns were recorded using a Miniflex diffractometer (Rigaku) with Cu Kα radiation, scanned over 5–45^o^ at a rate of 1^o^ min^-1^. Proton nuclear magnetic resonance (^1^H NMR) analysis was used to confirm the compositions of MOF-808 and MOF-808 EDTA. MOF samples (10 mg) were digested in a mixture of 30 μL HF and 570 μL DMSO‑*d*_6_ prior to measurement. Specific surface areas were measured using the Brunauer-Emmett-Teller (BET) method with an ASAP 2020 PLUS instrument under N_2_ atmosphere at 77 K. Samples were activated at 60 ^o^C under vacuum for 12 h before analysis. Porosity was determined gravimetrically using the equation: Porosity (%) = 100 × (W_wet_–W_dry_)/ρ_water_ ∙ V, where W_wet_ and W_dry_ are the wet and dry weights, ρ_water_ is the density of water at 25^o^C, and V is the sample volume. Tensile strength and elongation at break were measured using a universal testing machine (UTM, Autograph AGS-X Series, SHIMADZU) with a speed of 1 mm min^-1^ at room temperature. TGA was conducted using an SDT 650 (TA Instruments) under air. Samples were first dried at 100 ^o^C for 30 min, then heated from 100 ^o^C to 800 ^o^C at 10 ^o^C min^-1^ to determine MOF content. Chemical composition and molecular interactions were examined using attenuated total reflectance-Fourier transform infrared (ATR-FTIR, FT/IR-4700, JASCO) over 3500–650 cm^-1^ with a resolution of 4 cm^-1^ and 16 scans. The distribution of PAN and PVP in hollow fiber substrates was analyzed via X-ray photoelectron spectroscopy (XPS, PHI 5000 VersaProbe, Ulvac-PHI) using Al Kα radiation (1486.6 eV). Depth profiling was carried out by Ar^+^ ion etching (2.0 keV) for 0.1 min per cycle, proceeding from the outer to inner surface.

**Adsorption isotherm**

HPM 2nd EDTA (10 mg) was added to a conical tube containing 10 mL of heavy metal ion solutions at concentrations of 25, 50, 100, 300, 500, and 1000 ppm. The mixtures were shaken at 25 ^o^C for 24 h to reach adsorption equilibrium. Afterward, the samples were separated by filtration, and the filtrates were analyzed using inductively coupled plasma atomic emission spectroscopy (ICP-OES) to determine the residual metal concentrations.

A linearized Langmuir isotherm model was applied to estimate the maximum adsorption capacity and binding affinity of HPM 2nd EDTA for metal ions. The Langmuir model, which assumes monolayer adsorption on a homogeneous surface, is described by the following equations:

$q_{e}=\frac{q_{m}K_{L}C_{e}}{1+K_{L}C_{e}}$ Equation (S1)

$\frac{C_{e}}{q_{e}}=\frac{1}{K_{L}+q_{m}}+\frac{C_{e}}{q_{m}}$ Equation (S2)

where $q_{e}$ is the equilibrium adsorption capacity (mg g^-1^), $q_{m}$ is the maximum adsorption capacity (mg g^-1^), $K_{L}$ is the Langmuir constant related to binding affinity (L mg^-1^), and $C_{e}$ is the equilibrium concentration of metal ions in solution (mg L^-1^).

**Adsorption kinetics**

Samples (10 mg) were added to 10 mL of 300 ppm metal ion solution in conical tubes and shaken at 25 ^o^C. At predetermined time intervals (0.5 h, 1 h, 3 h, 6 h, 12 h, and 24 h), the samples were separated, and the supernatants were analyzed using ICP-OES. The adsorption kinetics were evaluated using the pseudo-second-order model, described by Equation (S3):

$\frac{t}{q_{t}}=\frac{1}{k_{2}q_{e}^{2}}+\frac{t}{q_{e}}$ Equation (S3)

where $q_{t}$ is the amount of metal ions adsorbed at time $t$ (mg g^-1^), $q_{e}$ is the adsorbed capacity at equilibrium (mg g^-1^), $k_{2}$ is the pseudo-second-order rate constant (mg mg^-1^ min^-1^), and $t$ is the contact time (min).

**Desorption of metal ions**

Regeneration of the adsorbent was performed by immersing the sample in a 0.1 M EDTA-2Na solution at 25 ^o^C for overnight.

**Module design and evaluation of dynamic metal ion capture**

Seven HPM 2nd EDTA hollow fibers (15 cm each) were assembled to create a fiber module with a total length of 105 cm. The module was used to assess dynamic adsorption performance with a mixed-metal ion solution containing 10 ppm each of Pb^2+^, Ni^2+^, and Co^2+^. The solution was continuously fed through the module at 0.5 bar, with a flow rate of 0.05 mL min^-1^ controlled by a downstream restrictor. Effluent was collected at regular time intervals, and the concentrations of metal ions were quantified by ICP-OES.

**Statistical Analysis**

Data are presented as mean ± standard deviation (SD). Sample sizes (n) are indicated in the figure legends. No formal statistical hypothesis testing was performed. Instead, descriptive statistics were used to illustrate reproducibility and variability.

**2. Supplementary Figures**


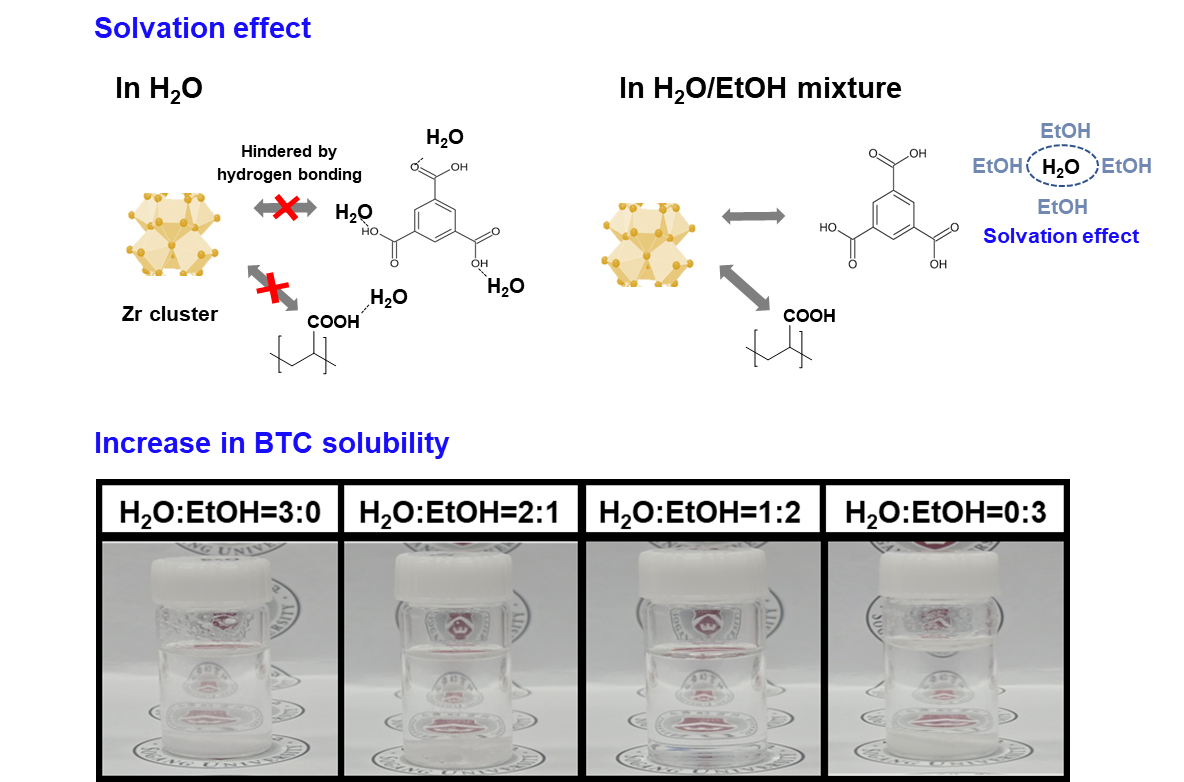


**Figure S1.** The effect of EtOH addition on the growth behavior of MOF-808.


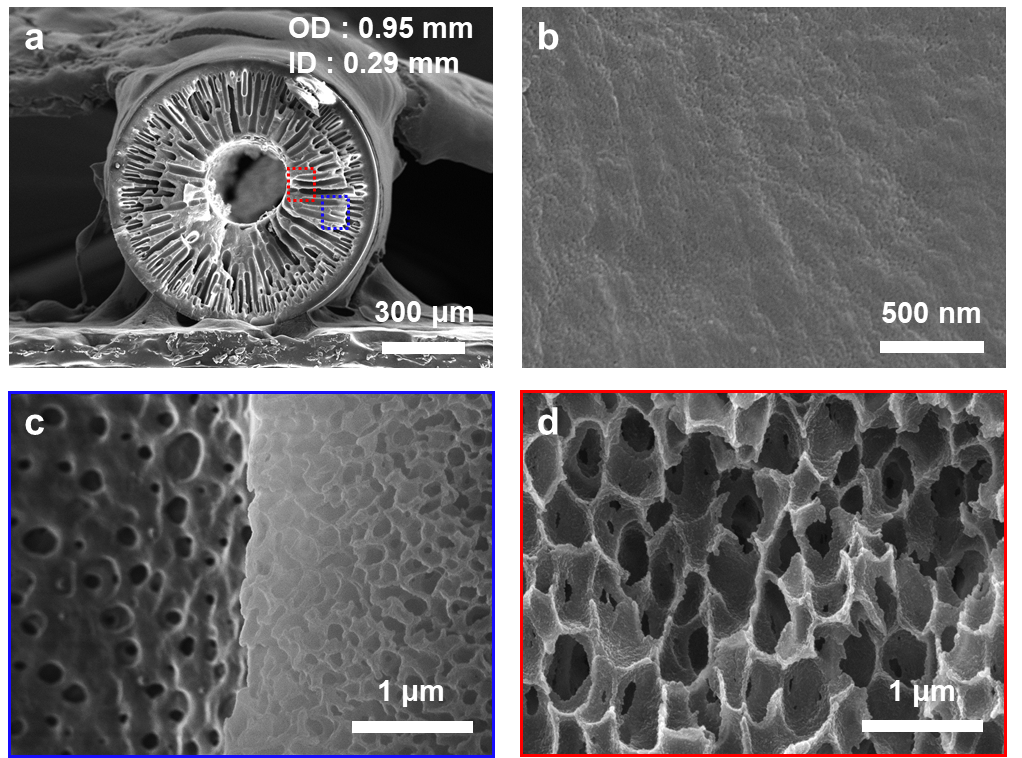


**Figure S2.** SEM images of PAN hollow fiber substrate: (a) overall cross-sectional view (b) outer surface (c) intermediate layer of the cross-section (d) inner layer of the cross-section).

**
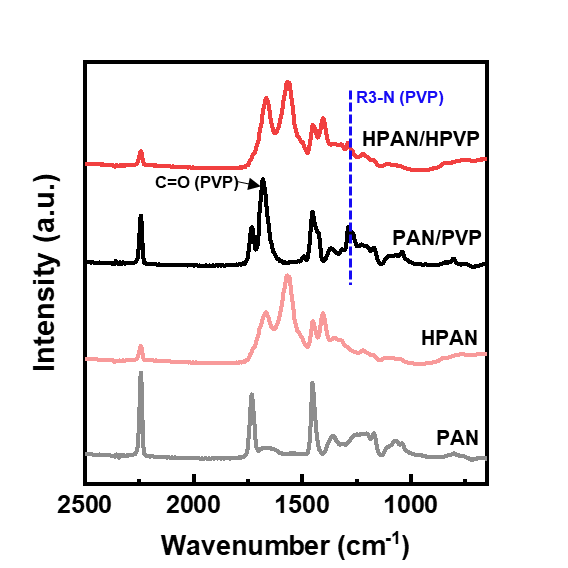
**

**Figure S3.** FT-IR spectra of FS-PAN, FS-PAN/PVP, FS-HPAN, and FS-HPAN/HPVP.

**Figure S4.** Weight drop ratio of 3 M HPAN after immersion in the solvent employed during the first growth step. Data are shown as mean ± SD (n = 3).

**Figure S5.** Porosity of 3 h HPAN as a function of NaOH concentration. Data are shown as mean ± SD (n = 3).

**Figure S6.** Tensile strength and elongation at break of 3 h HPAN as a function of NaOH concentration.


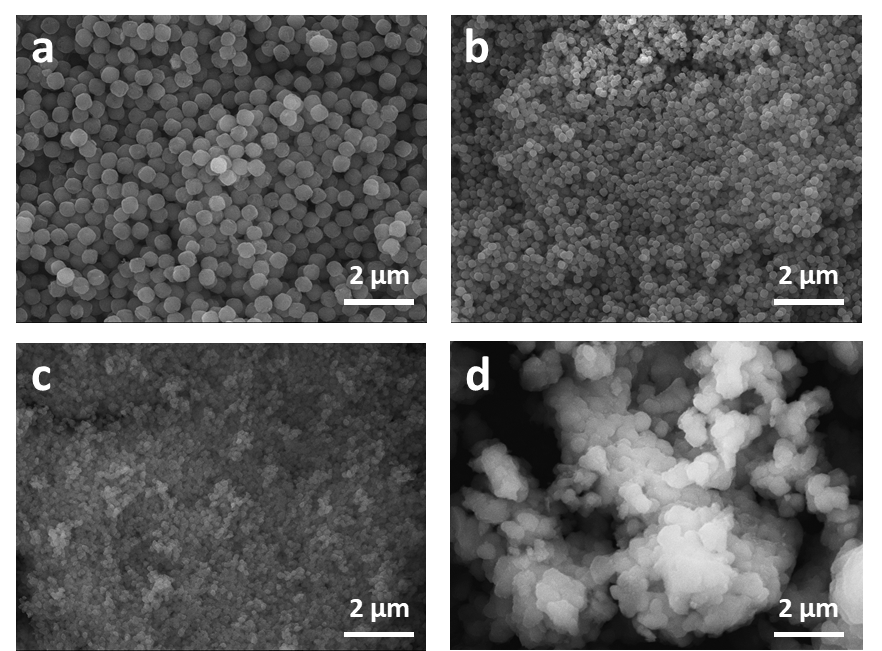


**Figure S7.** SEM images of bulk-synthesized MOF-808 with varying EtOH fractions in the H_2_O:EtOH mixture: (a) MOF-808 (9:0) (b) MOF-808 (7:2) (c) MOF-808 (5:4) (d) MOF-808 (4:5)


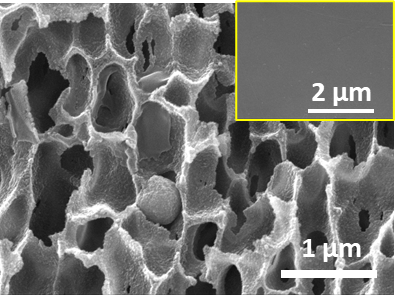


**Figure S8.** Cross-sectional SEM image of HPM 1st (9:0); the inset image shows the outer surface SEM image.

**Figure S9.** TGA curves of HPAN, HPAN-Zr, and HPM 1st (prepared with varying EtOH fractions in the H_2_O:EtOH mixture).

**
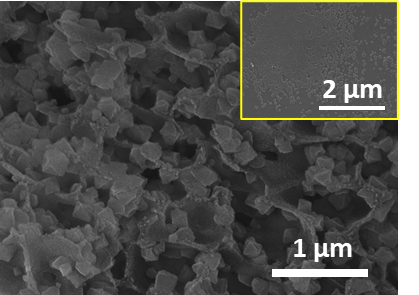
**

**Figure S10.** Cross-sectional SEM image of the HPM 2nd 48 h; the inset image shows the outer surface SEM image.

**Figure S11.** TGA curves of HPAN 2nd after secondary growth as a function of reaction time. MOF-808 loading was calculated using the equation: W_ZrO2_ × (M_MOF-808_/6M_ZrO2_), where W_ZrO2_ is the residual mass (%), M_MOF-808_ is the molecular weight of MOF-808 (g mol^-1^), and M_ZrO2_ is the molecular weight of ZrO_2_ (g mol^-1^).

**Figure S12.** TGA curves of PAN-Zr and HPAN-Zr.


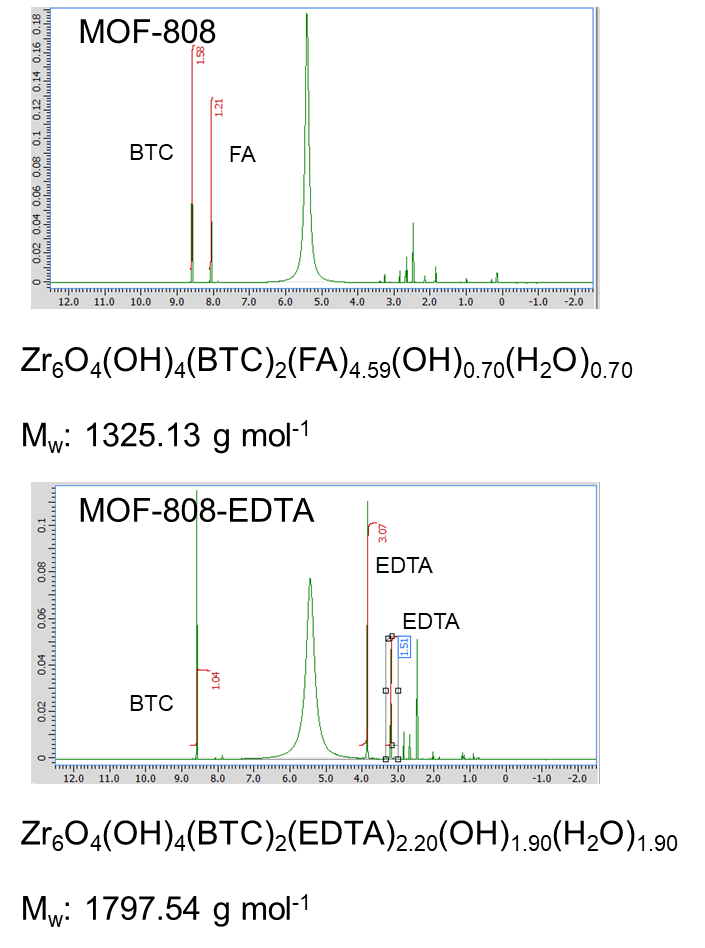


**Figure S13. ^1^**H NMR spectra and chemical compositions of bulk-synthesized MOF-808 and MOF-808 EDTA.

**Figure S14.** TGA curves of HPM 2nd EDTA before and after 1 h sonication.


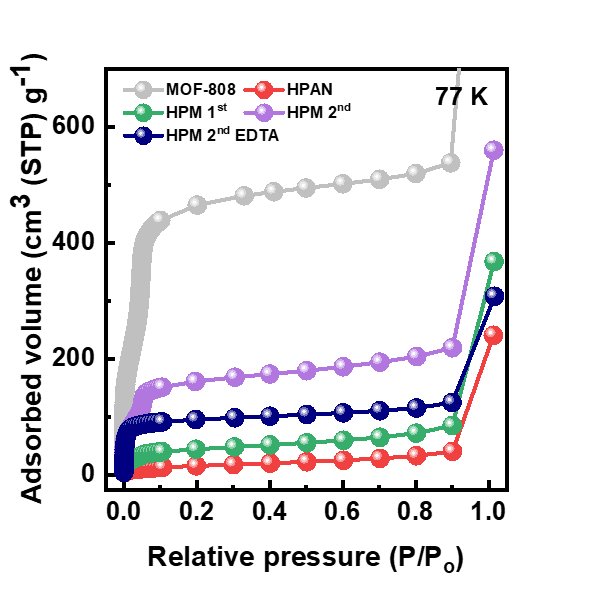


**Figure S15.** Nitrogen adsorption isotherms of bulk-synthesized MOF-808, HPAN, HPM 1st, HPM 2nd, and HPM 2nd EDTA (measured at 77 K).


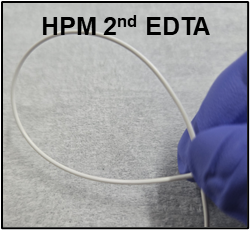


**Figure S16.** Photographic image of HPM 2nd EDTA under bending stress.


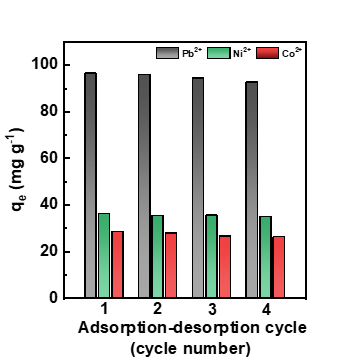


**Figure S17.** Metal ion adsorption capacity of HPM 2nd EDTA over multiple adsorption–desorption cycles in a 300 ppm metal ion solution at 25 ^o^C; desorption was performed using a 0.1 M EDTA-2Na solution.


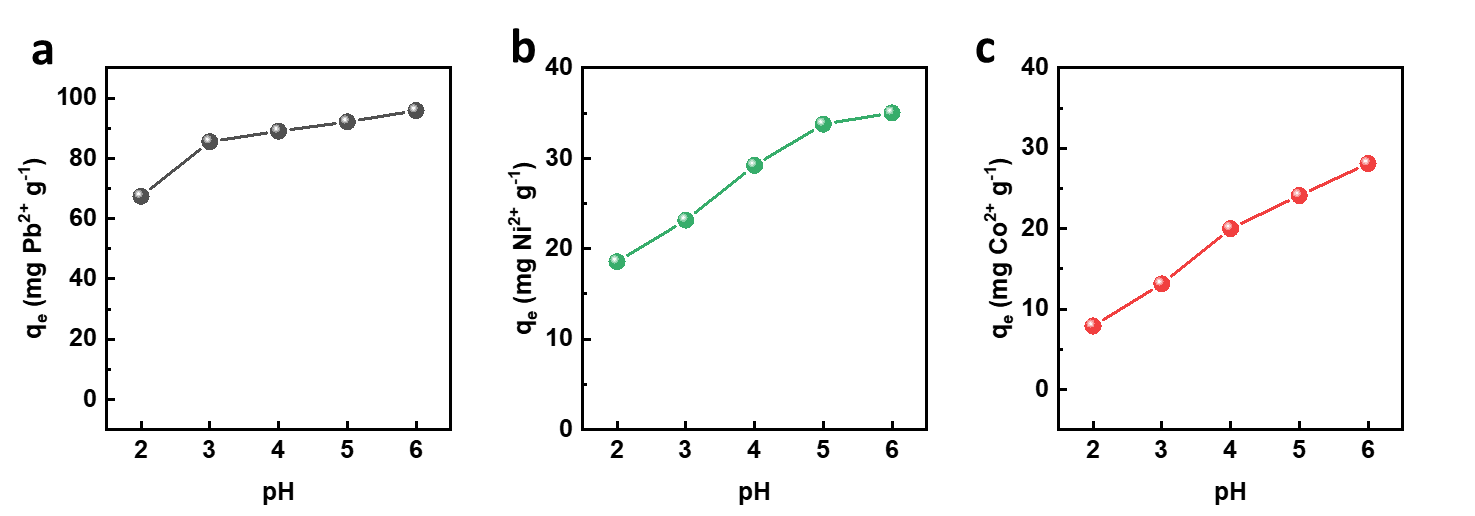


**Figure S18.** Metal ion uptake of HPM 2nd EDTA as a function of solution pH (300 ppm metal ion solution) for (a) Pb^2+^, (b) Ni^2+^, and (c) Co^2+^ at 25 ^o^C.


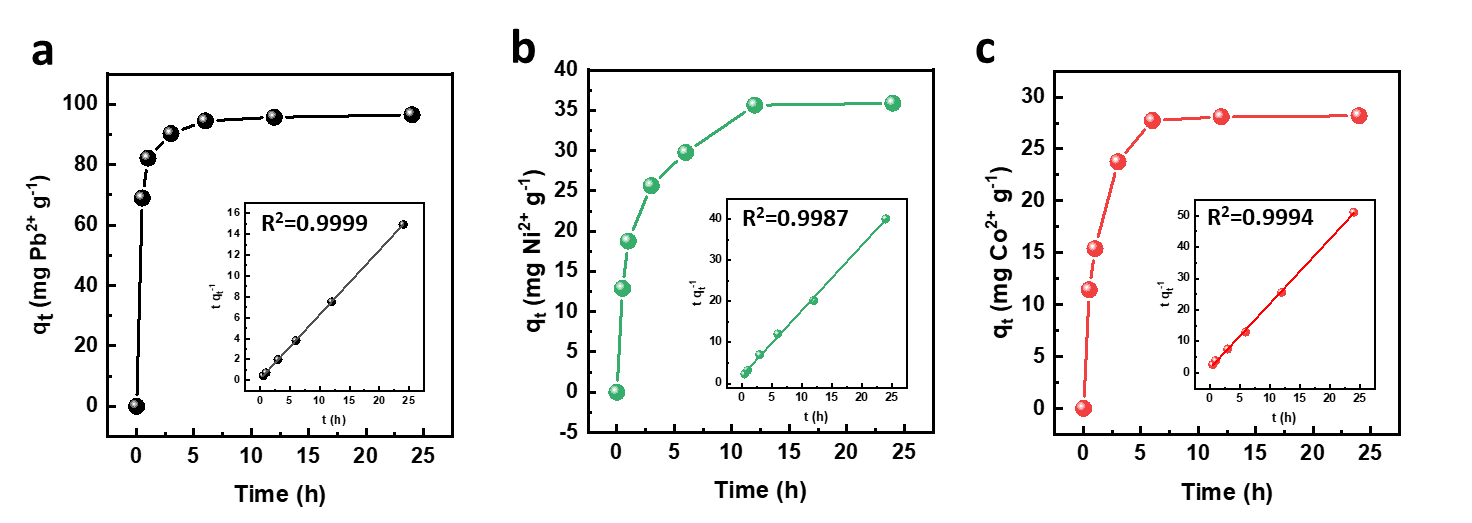


**Figure S19.** (a–c) Metal ion uptake of HPM 2nd EDTA over time from 300 ppm metal solutions for at 25 ^o^C (a) Pb^2+^, (b) Ni^2+^, and (c) Co^2+^. Inset images show the fitted curves based on the pseudo-second-order kinetic model.


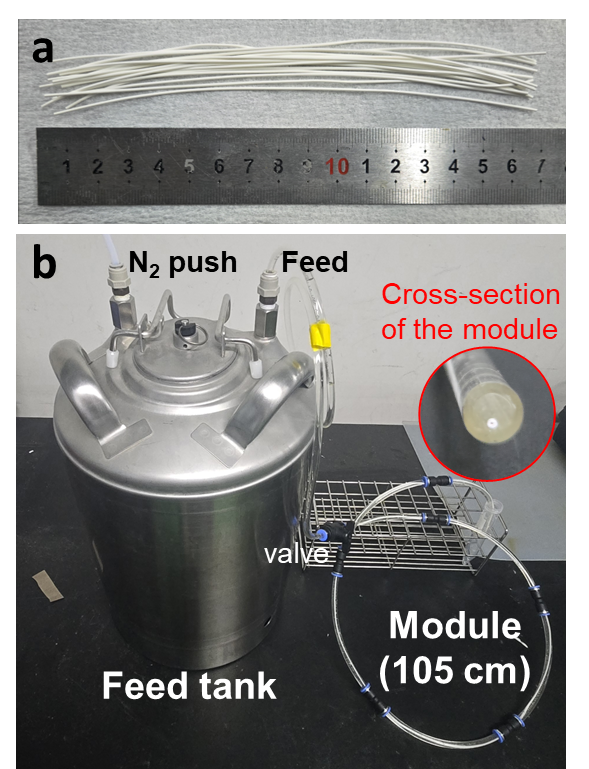


**Figure S20.** (a) Photograph of HPM 2nd EDTA used in the module and (b) experimental setup for dynamic metal ion capture.


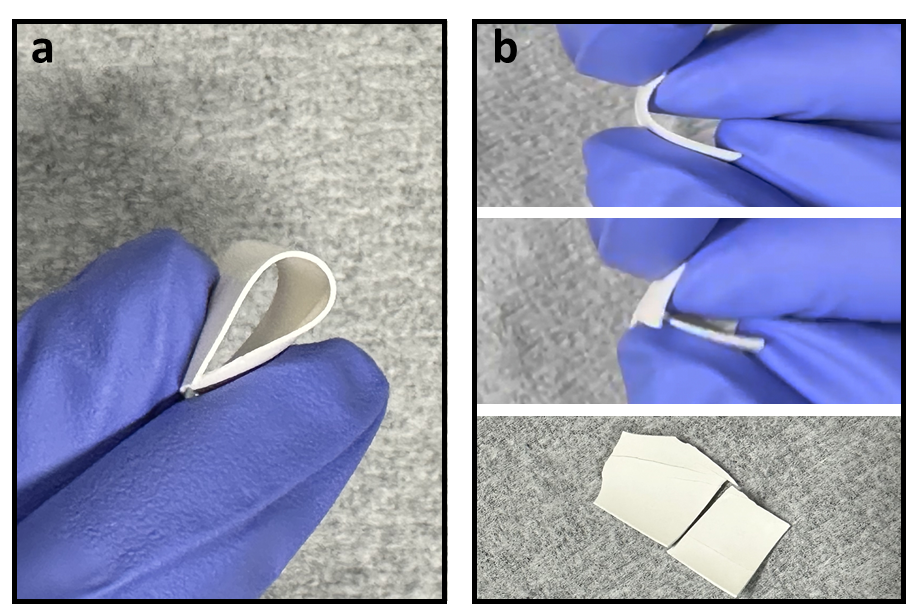


**Figure S21.** Photographic comparison of mechanical flexibility between (a) FS-HPAN/HPVPM 2nd EDTA and (b) PAN/PVP/M EDTA.

**3. Supplementary Tables**

**Table S1.** Spinning conditions for the fabrication of PAN hollow fiber substrates.

| **Spinning Parameters** | |
| --- | --- |
| Dope composition (wt.%) | PAN:PVP:DMSO = 18:7:75 |
| Dope flow rate (mL min^-1^) | 5 |
| Bore flow rate (mL min^-1^) | 0.5 |
| Dope temperature (^o^C) | 50 |
| Quench bath temperature (^o^C) | 25 |
| Air gap (cm) | 1 |

**Table S2.** XPS-derived chemical compositions of PAN hollow fiber substrate at different etching times.

| **Etching time (min)** | **C-C, C-H**  **C-N, C-O (%)** | **C≡N**  **(%)** | **C=O**  **(%)** | **PVP/PAN ratio**  **(%)** |
| --- | --- | --- | --- | --- |
| **0.1** | 86.3 | 6.9 | 6.8 | 49.5 |
| **0.3** | 86.3 | 10.9 | 2.9 | 21.0 |
| **0.6** | 88.1 | 11.7 | 0.2 | 1.5 |

* PVP/PAN ratios (%) = (A_C=O_/A_C≡N_) × 100, where A_C=O_ and A_C≡N_ are the area fractions of the C=O and C≡N peaks, respectively.

**Table S3.** Langmuir isotherm parameters for Pb^2+^, Ni^2+^, and Co^2+^ adsorption onto HPM 2nd EDTA.

|  | **Parameters from the Langmuir Linear Fitting** | | |
| --- | --- | --- | --- |
|  | $q_{m}$(mg g^-1^) | $K_{L}$ (L mg^-1^) | $R^{2}$ |
| **Pb^2+^** | 99.0 | 0.3507 | 0.9999 |
| **Ni^2+^** | 38.9 | 0.0883 | 0.9997 |
| **Co^2+^** | 29.5 | 0.0892 | 0.9999 |

**Table S4.** Pseudo-second-order kinetic parameters for the adsorption of Pb^2+^, Ni^2+^, and Co^2+^ onto HPM 2nd EDTA.

|  | **Parameters from the Pseudo-Second-Order Model** | | |
| --- | --- | --- | --- |
|  | $k_{2}$(mg mg^-1^min-1) | q_e,cal_  (mg g-^1^) | $R^{2}$ |
| **Pb^2+^** | 0.8514 | 97.1 | 0.9999 |
| **Ni^2+^** | 0.3801 | 37.7 | 0.9987 |
| **Co^2+^** | 0.8672 | 29.2 | 0.9994 |

**Table S5.** Pseudo-second-order kinetic parameters for Pb^2+^adsorption onto FS-HPAN/HPVPM 2nd EDTA and FS-PAN/PVP/M EDTA

|  | **Parameters from the Pseudo-Second-Order Model** | | |
| --- | --- | --- | --- |
|  | $k_{2}$(mg mg^-1^min-1) | q_e,cal_ | $R^{2}$ |
| **FS-HPAN/HPVPM 2nd EDTA** | 0.8175 | 86.2 | 0.9999 |
| **FS-PAN/PVP/M EDTA** | 0.3037 | 69.4 | 0.9983 |

**Table S6.** Thermogravimetric residue and corresponding MOF-808 loading for three replicates of HPM 1st and HPM 2nd, demonstrating the reproducibility of the in-situ and secondary MOF growth processes.

|  | **HPM 1st** | | **HPM 2nd** | |  |
| --- | --- | --- | --- | --- | --- |
|  | Residual Mass (%) | Calculated MOF-808 loading (%) | Residual Mass (%) | Calculated MOF-808 loading (%) | |
| **#1** | 13.6 | 24.4 | 19.2 | 34.4 | |
| **#2** | 13.2 | 25.6 | 20.4 | 36.7 | |
| **#3** | 14.3 | 24.9 | 19.9 | 35.8 | |
